# Supplementary material for: Protocol for an economic analysis of the randomised controlled trial of Improving the Well-being of people with Opioid Treated CHronic pain: I-WOTCH Study
Source: BMJ Open. 2020 Nov 20;10(11):e037243. doi: 10.1136/bmjopen-2020-037243 (PMC7682467; doi:10.1136/bmjopen-2020-037243)
Supplement: Supplementary data [file bmjopen-2020-037243supp001.pdf]

Supplementary Material

Resource use and costing for the I-WOTCH Intervention

**Table A1. Resource use and costs related to running I-WOTCH intervention**		
**I-WOTCH intervention running costs**		
Category	***Resource use***	***Unit cost***
Number of participants	Number of participants enrolled in the course	
Facilitator costs	Total number and duration of sessions per facilitator	Fixed fee per session (pension and national Insurance contributions included). Alternatively, grade (if NHS) or salary
Facilitator travel costs	Total number and type (i.e. car, train, etc.) of journeys	Train or bus fare and fixed fee per mile.
Administrator costs	Number of days / months dedicated to coordinate implementation of I-WOTCH intervention	Salary used to estimate fixed daily rate (pension and national insurance contributions included)
Facility costs	Number of days facility has been hired per course delivered	Daily rate for venue
Hospitality costs	Food and drink	
Consumables	Stationary (e.g. letters, stamps, notebooks, pens, pencils, etc.)	
Course materials costs		
My Opioid Manager Manual	Total number of manuals produced and delivered	Total printing costs and copy right fee
Relaxation Package	Total number of CDs produced and delivered	Total cost of CDs

Table A2. Resource use and costs related to training related to I-WOTCH intervention		
**Training costs**		
Category	***Resource use***	***Unit cost***
Trainer costs	Number of days / months - per trainer - dedicated to train facilitators to deliver I-WOTCH intervention	Fixed daily rate
Trainer travel cost	Total number and type (i.e. car, train, etc.) of journeys	Train or bus fare and fixed fee per mile
Facilitator costs	Total number and duration of sessions per facilitator	Fixed fee per session (pension and national insurance contributions included) or salary
Facilitator travel costs	Total number and type (i.e. car, train, etc.) of journeys	Train or bus fee and fixed fee per mile
Administrator costs	Number of days / months dedicated to coordinate implementation of I-WOTCH intervention	Salary used to estimate fixed daily rate (pension and national insurance contributions included)
Facility costs	Number of days facility has been hired per course delivered	Daily rate for venue
Hospitality costs		
Consumables		
Course materials costs		
My Opioid Manager Manual	Total number of manuals produced and delivered	Total printing costs
Relaxation Package	Total number of CDs produced and delivered	Total cost of CDs

Table A3. Data collection form used to characterise the I-WOTCH intervention facilitators	
**Characteristics of course facilitators**	
Variable	***Description***
Facilitator ID	Define unique key
Site	
Age	In years
Gender	
Ethnicity	Same categories used for participants
Type	Healthcare provider<br>Lay person
Profession / Experience	
Years of professional experience (for health care professional (HCP)) or facilitation (lay person)	
Number of curses facilitated	

Table A4. Data collection form used to characterise the course in I-WOTCH intervention	
**Course venues, facilitators, and participants**	
Variable	***Description***
Course ID	
Venue	
Facilitator	Facilitator profession/experience
Number enrolled	
Attendance	Attendance per patient per session
