# Supplementary material for: Protocol for an economic analysis of the randomised controlled trial of Improving the Well-being of people with Opioid Treated CHronic pain: I-WOTCH Study
Source: BMJ Open. 2020 Nov 20;10(11):e037243. doi: 10.1136/bmjopen-2020-037243 (PMC7682467; doi:10.1136/bmjopen-2020-037243)
Supplement: Author's manuscript [file bmjopen-2020-037243.draft_revisions.pdf]

BMJ Open is committed to open peer review. As part of this commitment we make the peer review history of every article we publish publicly available.

When an article is published we post the peer reviewers' comments and the authors' responses online. We also post the versions of the paper that were used during peer review. These are the versions that the peer review comments apply to.

The versions of the paper that follow are the versions that were submitted during the peer review process. They are not the versions of record or the final published versions. They should not be cited or distributed as the published version of this manuscript.

BMJ Open is an open access journal and the full, final, typeset and author-corrected version of record of the manuscript is available on our site with no access controls, subscription charges or pay-per-view fees (http://bmjopen.bmj.com).

If you have any questions on BMJ Open's open peer review process please email info.bmjopen@bmj.com

BMJ Open

Protocol for an economic analysis of the randomised controlled trial of improving the wellbeing of people with opioid treated chronic pain: I-WOTCH study

Journal:	*BMJ Open*
Manuscript ID	bmjopen-2020-037243
Article Type:	Protocol
Date Submitted by the Author:	24-Jan-2020
Complete List of Authors:	Manchira Krishnan, Sheeja; University of York, Department of Health Sciences<br>Gc, Vijay; University of York Centre for Health Economics, ; University of East Anglia Norwich Medical School,<br>Sandhu, Harbinder; University of Warwick Warwick Medical School, Clinical Trials Unit<br>Underwood, Martin; University of Warwick, Warwick Clinical Trials Unit, Warwick Medical School; University Hospitals Coventry and Warwickshire NHS Trust<br>Eldabe, Sam; James Cook University Hospital, Pain Department<br>Manca, Andrea; University of York, Center for Health Economics<br>Iglesias, C; University of York, Department of Health Sciences
Keywords:	HEALTH ECONOMICS, PAIN MANAGEMENT, Protocols & guidelines < HEALTH SERVICES ADMINISTRATION & MANAGEMENT

SCHOLARONE™
Manuscripts

I, the Submitting Author has the right to grant and does grant on behalf of all authors of the Work (as defined in the below author licence), an exclusive licence and/or a non-exclusive licence for contributions from authors who are: i) UK Crown employees; ii) where BMJ has agreed a CC-BY licence shall apply, and/or iii) in accordance with the terms applicable for US Federal Government officers or employees acting as part of their official duties; on a worldwide, perpetual, irrevocable, royalty-free basis to BMJ Publishing Group Ltd ("BMJ") its licensees and where the relevant Journal is co-owned by BMJ to the co-owners of the Journal, to publish the Work in this journal and any other BMJ products and to exploit all rights, as set out in our licence.

The Submitting Author accepts and understands that any supply made under these terms is made by BMJ to the Submitting Author unless you are acting as an employee on behalf of your employer or a postgraduate student of an affiliated institution which is paying any applicable article publishing charge ("APC") for Open Access articles. Where the Submitting Author wishes to make the Work available on an Open Access basis (and intends to pay the relevant APC), the terms of reuse of such Open Access shall be governed by a Creative Commons licence – details of these licences and which Creative Commons licence will apply to this Work are set out in our licence referred to above.

Other than as permitted in any relevant BMJ Author's Self Archiving Policies, I confirm this Work has not been accepted for publication elsewhere, is not being considered for publication elsewhere and does not duplicate material already published. I confirm all authors consent to publication of this Work and authorise the granting of this licence.

For peer review only

Protocol for an economic analysis of the randomised controlled trial of improving the wellbeing of people with opioid treated chronic pain: I-WOTCH study

Sheeja Manchira Krishnan[1], Vijay S. Gc[2], Harbinder K Sandhu[3], Martin Underwood[3,4], Sam Eldabe[5], Andrea Manca[2], Cynthia P Iglesias Urrutia[1] on behalf of the I-WOTCH team

[1] Department of Health Sciences, University of York, Heslington, York, YO10 5DD, UK

[2] Centre for Health Economics, University of York, Heslington, York, YO10 5DD, UK

[3] Warwick Clinical Trials Unit, Warwick Medical School, University of Warwick, Gibbet Hill Campus, Coventry, CV4 7AL, UK

[4] University Hospitals of Coventry and Warwickshire, Clifford Bridge Road, Coventry CV2 2DX

[5] Pain Department, The James Cook University Hospital, Marton Road, Middlesbrough, TS4 3BW, UK

Correspondence:

Dr. Cynthia P Iglesias Urrutia

Department of Health Sciences

University of York

Heslington

York YO10 5DD, UK

Email: cynthia.iglesias@york.ac.uk

Phone: 01904 32 1820

Keywords: Health economics analysis plan, with-in trial analysis, model based analysis, cost-effectiveness analysis, opioid tapering

Word count: 3769 (excluding title page, abstract, acknowledgements, funding statement, author contributions, and references)

Number of tables and figures: Figures 1, Tables 2

For peer review only - http://bmjopen.bmj.com/site/about/guidelines.xhtml

ABSTRACT

Introduction: The use of opioids for the treatment of chronic pain in England has steadily increased over the last two decades, despite lack of evidence for long-term effectiveness in pain relief, and significant, well documented physical and mental adverse events associated with their long-term use. Guidelines recommend tapering when harms outweigh benefits, but the addictive nature of opioids hinders simple dose reduction strategies. Improving the Wellbeing of people with Opioid Treated CHronic pain (I-WOTCH) trial tests a multicomponent self-management intervention aimed to help patients with chronic non-malignant pain taper their opioid doses.

Objective: Outline the methods to be used for the economic analysis of the I-WOTCH intervention compared to best usual care.

Methods and analysis: Economic evaluation alongside the I-WOTCH study, prospectively designed to identify, measure, and value key healthcare resource use and outcomes arising from the treatment strategies being compared. A within-trial cost consequences analysis and a model-based long-term cost-effectiveness analysis will be conducted from the UK National Health Service and Personal Social Service perspective. The former will quantify key parameters to be used in a Markov model designed to estimate the long-term cost-effectiveness of the I-WOTCH programme against best usual care. Regression equations will be used to derive parameters such as transition probabilities, utilities, and costs associated with the model's states and events. Probabilistic sensitivity analysis will be used to assess the impact of parameter uncertainty onto the predicted costs and health outcomes, and the resulting value for money assessment of the I-WOTCH programme.

Ethics: Full ethics approval was granted by Yorkshire & The Humber – South Yorkshire Research Ethics Committee on September 13th, 2016 (16/YH/0325). Local approvals were sought for each area in which recruitment was undertaken. Current protocol: version 1.7, date 31 July 2019.

Trial number: International Standard Randomised Controlled Trial Number: 49470934 (6 Feb 2017).

Word count: 300 words

For peer review only

Article summary

Strengths and limitation of this study:

- First economic evaluation of a complex, public health intervention directed towards improving the wellbeing of opioid treated patients with non-malignant chronic pain in the UK setting.
- The planned data collection for the purpose of economic evaluation alongside the trial will aid the estimation of the key parameters to assess the value for money of I-WOTCH
- The economic analysis will provide decision makers with valuable evidence for potential implementation of the self-management support program aimed at opioid tapering
- Uncertainty may remain as to the long-term effectiveness and cost-effectiveness of the I-WOTCH programme due to the limited follow-up duration of the study.

For peer review only

1. BACKGROUND

Nearly half of the UK adult population (43%) is living with chronic pain (i.e. pain lasting >3 months), the prevalence of which increases with age.[1] Opioids are commonly prescribed for chronic pain and a recent study reports an increase in the number of opioid prescriptions (~34%) in England between 1998 and 2016.[2] Analysis of prescription data shows that after this long increasing trend, there is a slight decrease in the annual number of opioid pain medicines since 2016.[3] Despite the demonstrated short-term effectiveness of opioids, evidence of their long-term impact in terms of pain relief and improvement in functional status is scant.[4,5] This situation is compounded by concerns over the fact that long-term use of opioids can lead to adverse events (at an estimated absolute rate of 78%) affecting the respiratory, cardiovascular, gastrointestinal, and central nervous systems.[6] These include common adverse events such as dry mouth, nausea, and constipation as well as more serious adverse events including sleep-disordered breathing, respiratory depression, and opioid-related deaths (at an absolute rate of 7.5%).[6-8] Equally concerning is the fact that opioids use is also associated with mental health and anxiety disorders, major depression, and dysthymia.[9] Their long-term use may cause problematic patterns of substances use, leading to substances overuse.[10] Risk factors for opioid-related adverse events include older age, higher doses of this class of drugs and their long-term use.[11,12]

Clinical guidelines for prescribing opioids in chronic pain recommend tapering when the possible harms from their use may outweigh any expected benefits [13] and yet, to our knowledge, no validated protocol or intervention exist to help patients reduce their opioid doses and manage their chronic pain.[14] A number of studies have evaluated interventions (e.g. acupuncture, ketamine assisted dose reduction, and behavioural strategies such as motivational interviewing, psychiatric consultation, cognitive behavioural therapy, and mindfulness) that support opioid tapering.[15-18] The conclusions drawn by these studies are limited by the quality of their design or insufficient follow-up period.[14] Relevant for our study, there are no existing economic evaluations of opioid tapering strategies in the management of chronic non-malignant pain.

The Improving the Well-being of People with Opioid Treated Chronic Pain (I-WOTCH) is an NIHR funded randomised controlled trial (RCT) evaluating the effectiveness and cost-effectiveness of a patient-centred, multicomponent self-management intervention targeting withdrawal of strong opioids among those living with chronic non-malignant pain.[19] The I-WOTCH trial is designed to help people to reduce their opioid consumption, manage pain interference, and enhance their quality of life.

This paper describes the protocol for the economic analysis that has been designed as an integral part of the I-WOTCH trial.

2. METHODS

2.1 Study details (population, setting, location, intervention and comparator)

A detailed study protocol for the I-WOTCH trial has been reported in a separate manuscript.[19] Briefly, I-WOTCH is a multi-site, patient-centred, open randomised controlled

For peer review only - http://bmjopen.bmj.com/site/about/guidelines.xhtml

trial enrolling adult patients with non-malignant chronic pain in England. The trial's target sample size is 542 participants, individually randomised (1:1) to receive the I-WOTCH intervention or best usual care. The I-WOTCH intervention is an 8-10 weeks course -consisting of a mixture of group sessions led by two trained I-WOTCH facilitators and two one-to-one individual sessions and two telephone consultations with the I-WOTCH trained nurse facilitator - adjunct to best usual care. Best usual care consists of general practitioner (GP) care with relaxation package and a booklet called 'My Opioid Manager'.

2.2 Type of economic evaluation (cost-consequences and cost-effectiveness analyses)

Two types of economic analysis will be conducted: a within-trial cost-consequences analysis (CCA) and a long-term model based cost-effectiveness analysis (CEA), the details of which are provided in section 3.

2.3 Study perspective

The perspective for both the CCA and CEA will be that of the National Health Service (NHS) and Personal Social Service for England and Wales.

2.4 Time horizon

The CCA and CEA will adopt a 12-month and a patients' life time horizons, respectively.

2.5 Discount rate

Estimates of mean cost and health benefits observed during the 12-month trial follow up period will not be discounted (for the purpose of the CCA), while those predicted to accrue beyond the study follow-up (for the purpose of the CEA) will be discounted using a 3.5% annual discount rate as per National Institute for Health and Care Excellence (NICE) guidelines.[20]

2.6 Identification, measurements, and valuation of health outcomes

Table 1 describes the variables of interest to the economic analyses of I-WOTCH, the frequency of data collection, and data sources.

Table 1 Data collection strategy for I-WOTCH's economic analyses

Data collected	Source	Time of collection
Baseline data		
Age	PtQ	Baseline
Gender	PtQ	Baseline
Ethnic group	PtQ	Baseline
Current work status	PtQ	Baseline
Age at leaving full time education	PtQ	Baseline
Pain duration	PtQ	Baseline

For peer review only - http://bmjopen.bmj.com/site/about/guidelines.xhtml

Opioid intake duration	PtQ	Baseline
Pain conditions	PtQ<br>GPRs	Baseline<br>At 12 months only
Pain severity stratification group	PtQ	Baseline
Measures of health benefit		
Activities of Daily Living (PROMIS PI-SF-8A)	PtQ	Baseline, 4, 8 and 12 months
Severity of opioid withdrawal symptoms (ShOWS)	PtQ<br>PtD	Baseline, 4, 8 and 12 months<br>Weekly over first 4 months
Generic health related quality of life (EQ-5D-5L)	PtQ<br>PtD	Baseline, 4, 8 and 12 months<br>Weekly over first 4 months
Resource use (volume, admissions, consultations, attendances and/or contacts)		
Medications	PtQ<br>GPRs	Baseline, 4, 8 and 12 months<br>At 12 months only
Hospital	PtQ<br>GPRs	Baseline, 4, 8 and 12 months<br>At 12 months only
Hospital outpatient	PtQ<br>GPRs	Baseline, 4, 8 and 12 months<br>At 12 months only
GP surgery	PtQ<br>GPRs	Baseline, 4, 8 and 12 months<br>At 12 months only
GP home	PtQ<br>GPRs	Baseline, 4, 8 and 12 months<br>At 12 months only
GP telephone	GPRs	At 12 months only
Practice nurse	PtQ<br>GPRs	Baseline, 4, 8 and 12 months<br>At 12 months only
Practise nurse telephone	GPRs	At 12 months only
District nurse (i.e. at home)	PtQ<br>GPRs	Baseline, 4, 8 and 12 months<br>At 12 months only
NHS 111	GPRs	At 12 months only
Occupational therapist	PtQ	Baseline, 4, 8 and 12 months
Counsellor	PtQ	Baseline, 4, 8 and 12 months
Psychologist	PtQ	Baseline, 4, 8 and 12 months
Social worker	PtQ	Baseline, 4, 8 and 12 months
Physiotherapist	PtQ	Baseline, 4, 8 and 12 months
Referrals	GPRs	At 12 months only
Investigations	GPRs	At 12 months only
Ambulance incidents	GPRs	At 12 months only
Accident and emergency	GPRs	At 12 months only
Other	PtQ<br>GPRs	Baseline, 4, 8 and 12 months<br>At 12 months only
Key for Table 1: GPRs - GP records, PtD - Patient diaries, and PtQ - Patient questionnaire		

For peer review only

The CCA will consider the following three health outcome measures over the 12-month trial follow-up period:

1) *Activities of Daily Living* (ADL) - measured using the patient reported outcomes measurement information system pain intensity short form-8A questionnaire (PROMIS-PI-SF-8A);[21]
2) *Severity of opioid withdrawal symptoms* - measured using the short opiate withdrawal scale (ShOWS);[22]
3) *EuroQol five dimensions* instrument - a preference-based generic measure of health related quality of life typically used in healthcare economic evaluation.[23,24]

The PROMIS-PI-SF-8A is a composite of eight questions rated on a scale of 1-5 that aim to measure the degree of interference of pain on day-to-day activities, work at home, ability to participate in social activities, household chores, fun activities, enjoyment of social activities, life and family life. The total raw score is the sum of individual responses to each question thus forming a maximum score of forty and a minimum score of eight. Higher scores reflect large interference or change in participant's ability to perform daily activities. This instrument supports calculation of a common metric and will be converted to a T score.[21]

ShOWS identifies the severity of opiate withdrawal symptoms on ten different categories namely: feeling sick, stomach cramps, muscle spasms, feeling of coldness, heart pounding, muscular tension, aches and pains, yawning, runny eyes, and insomnia. The response to each question is attributed to an individual score of 0 to 3, with 0 indicating no symptoms, 1 mild, 2 moderate, and 3 severe.[22] Higher overall scores (estimated as the sum of all individual scores with a maximum score of thirty and a minimum score of zero) indicate higher severity of opioid withdrawal symptoms.

The EQ-5D-5L questionnaire [23] describes health in five domains (mobility, self-care, usual activities, pain or discomfort, and anxiety or depression). Each domain has five levels of severity (no problems/slight problems/moderate problems/severe problems/unable to do). Responses to each domain range from one to five and an individual response consists of a five-digit number representing the individual's response to all five domains. Combinations of the five domains describe 3125 states. A set of index scores to calculate utility values for the EQ-5D-5L have been published recently [25] but are still subject to methodological controvercy. Until these are resolved, current NICE's recommendation is to convert EQ-5D-5L responses onto the EQ-5D-3L scale [24] using the mapping function developed by van Hout *et al* [26] which is what we shall do in our study.

The CEA will integrate predicted survival and EQ-5D (for each model state and clinical event) to derive an estimate of quality-adjusted life years (QALYs) - the health benefit of choice in our CEA - under the I-WOTCH and best usual care strategies.

2.7. Identification, measurement and valuation of resource use

As described in Table 1, patient-level resource use data are collected in the trial and will be complemented by information routinely collected in GP records. Costing will be carried out in UK pound sterling at 2018 prices. Unit cost for tests, investigations, inpatient hospital admissions and day care procedures will be estimated using National Reference Costs and

For peer review only - http://bmjopen.bmj.com/site/about/guidelines.xhtml

Healthcare Resource Group (HRG) codes.[27] Referrals and consultations will be costed using Personal and Social Services Research Unit (PSSRU) statistics.[28] If necessary, we will use unit costs from previous versions of the PSSRU report [29,30] and inflate them to the year 2018 using inflation and price indices from the Office of National Statistics (ONS).[31] Costs associated with usual care will include the cost of the relaxation CD (i.e. printing and production costs) and 'My Opioid Manager'.

2.7.1 Microcosting of I-WOTCH intervention

A microcosting of the resources required to provide the I-WOTCH intervention will be conducted. A detailed description of the categories of resource use to be considered, their associated unit costs and the data collection forms used in the I-WOTCH microcosting exercise is provided in the Supplementary Material (Annex A Tables A1 - A4). Salary, facility, and travel costs will be assumed to be independent of the number of participants. Salaries for the facilitators and trainers will be estimated based on their average daily salary by grade. The facility costs will be calculated based on the number of venues hired, number of days hired, and daily venue hire rate. Travel costs will be considered as fixed cost per mile. Unit costs for nurses' time for face-to-face and telephone consultations will be extracted from the PSSRU 2018.[28] A per participant, locality, and course cost of I-WOTCH intervention will be reported.

2.7.2 Cost of medication

Unit costs of the medications (Table 1) will be obtained from the British National Formulary (BNF).[32] For each strength and preparation of a given pain killer drug, we will extract the cost per pack (or bottle) from the BNF and we will calculate the relevant morphine equivalent dose (MED) using the same algorithm used in the I-WOTCH clinical analysis.[32] This is being updated from that used to estimate MED for stratification at the time of randomisation. For each opioid-based medication reported to have been used by individuals in the I-WOTCH study we will estimate their unit cost per MED and use it to estimate a weighted average cost per MED over all opiod medications used in the trial.

2.8 Modelling

A Markov state-transition model has developed to facilitate the estimation of the long-term mean costs and QALYs associated with the I-WOTCH intervention and usual care. The initial conceptual structure of the model was informed by a systematic analysis of the components of I-WOTCH intervention. This task was supported and enhanced by critically appraising published decision models that evaluated the use of opioids in chronic non-malignant pain. The face validity of the model structure was further refined by holding a series of meetings with the project team involving clinical experts from the I-WOTCH study. This process is described in detail in a separate related manuscript currently in preparation.

Figure 1 depicts a schematic representation of our final model structure, which is organised around five key states (i.e. represented as ovals in Figure 1). At any time period, patients can be in one of the following mutually exclusive states:

For peer review only - http://bmjopen.bmj.com/site/about/guidelines.xhtml

a) long term opioid therapy (LTOT) - representing individuals candidate for the intervention, who have been using strong opioids for more than 3 months;
b) I-WOTCH tapering (IT) - individuals committed to tapering as part of the I-WOTCH intervention;
c) patient driven tapering (PT) - individuals self-committed to tapering without the intervention;
d) successful opioid-less management of pain (SOLMP) - individuals completed withdrawal from opioid use; and
e) dead (D).

Each state is associated with an average cost and utility score, derived using the methods described in section 3.12. Arrow's spearheads in Figure 1 represent the direction of allowed transitions per cycle. LTOT is the starting state for all patients in the I-WOTCH trial. Individuals in LTOT can engage with IT only once but are able to initiate PT at their will. Whilst they remain in the LTOT state, individuals can experience opioid related transient/emergent adverse events (TEAE) or persistent/serious adverse events (PSAE). Occasionally, adverse events may trigger a transition from LTOT to PT. Individuals in IT or PT may withdraw from opioid tapering at any point in time and go back to LTOT. Individuals in IT or PT can transit to SOLMP state only when they are no longer taking any opioids. Finally, individuals are at risk of death at any point in time (i.e. transitions into the absorbing dead state are allowed from the LTOT, IT, PT, and SOLMP states). A paper describing a detailed description of the conceptualization of the model, health states definition, clinical events, and associated transitions is in preparation. To maximise the use of the data collected during the trial and to model the events and transitions observed during the treatment and follow up period the state transion model uses a weekly cycle length during the first 4 months and monthly cycles beyond that.

3. Analysis

Statistical analysis for the CCA and CEA will be conducted using I-WOTCH's individual patient data (IPD) collected on resource use and outcomes, analysed on an intention to treat (ITT) basis. Statistical analysis and decision models are implemented in R.[33]

3.1 Within-trial cost-consequences analysis

The CCA will focus on estimating and reporting mean health outcomes, resource use, and costs by treatment arm at each follow up interval. No incremental analysis will be conducted. Total costs will be the sum of the costs of the healthcare resource items (described in Table 1) that patients used during the study period, plus the cost of either the I-WOTCH intervention or the usual care. Mean health outcomes will include those described in section 2.6 and reported at each follow up period by treatment arm.

3.1.1 Handling missing data

We will assess the extent of missing data in the patient-level costs and health outcomes collected during the 12 months follow-up of the I-WOTCH study and apply appropriate methods to handle the issue following the reccommendations by Faria *et al* .[34].

For peer review only - http://bmjopen.bmj.com/site/about/guidelines.xhtml

3.1.2 Statistical analysis

Descriptive statistics (i.e. mean, standard deviation (SD), lower and upper quartiles, minimum and maximum values) for all continuous variables reported in Table 1, plus total costs will be estimated by trial arm. Histograms and/or box-plots will be used to represent graphically these variables. Binary and categorical variables will be represented in terms of percentages. The impact of patient's baseline characteristics (i.e. age, gender, pain severity, opioid use, duration of opioid intake, and opioid medication related pain condition) as predictors of outcomes will be explored in a series of regression models fitted to ADL, ShOWS, EQ-5D-3L utility scores and cost data to inform the estimation of the Markov model's input parameters.

We will map EQ-5D-5L utility scores into EQ-5D-3L utility scores.[24,26] To account for idiosyncrasies of the EQ-5D-3L, i.e. multimodality, truncated support, and left-skewness, we will use a series of regression models (including mixture of beta models, adjusted limited dependent variable mixture models, two-part models). Each model goodness of fit and assessment of their predictive ability by carried out using the methods recommended by Hernandez-Alava et al.[35,36]

Many methods have been used to analyse cost data.[37] We will use generalised linear models (GLM) (with gaussian or gamma distributed errors and identity or log links) and where necessary two-part extensions of these models to account any mass at zero, the right skewed nature of the dependent variable and possible heteroskedasticity. Should administrative censoring (due to patients differential follow up) be present, the analysis will use inverse probability weighting (IPW) methods alongside our cost regression models.[38]

All regression models will explore the impact of the patient's baseline characteristics included in Table 1, and use these results to explore the role of patients's heterogeneity onto the cost-effectiveness results (more in section 3.2.3).

3.1.3 Sensitivity analysis

For the purposes of the CCA, we will carry out a per protocol analysis and compare the results with the intention to treat analysis. We will calculate the minimum vs actual number of group sessions needed to provide the I-WOTCH intervention to all participants in the intervention group. We will also consider a minimum and maximum number of participants per course observed in the I-WOTCH trial. We will use a one-way or threshold analyses on the assumptions made on estimating the costs.

3.2 Long-term model based cost-effectiveness analysis

3.2.1 Populating model parameters

Table 2 provides a list of key model parameters and the source for initial values, i.e. mean, SD or confidence interval. To estimate all model initial values we will use IPD from the I-WOTCH and GP records as well as any relevant publicly available evidence. Transitions from all model states to death will be extracted from age and sex adjusted all-cause mortality statistics from the ONS.[39] Transition probabilities between all the other model states will be derived using risk equations regression models (e.g. time-to-event and / or logit regression models

For peer review only - http://bmjopen.bmj.com/site/about/guidelines.xhtml

depending on the nature of the transition). Costs and utilities parameters for each of the model states and events will be estimated using the regression models described in 3.1.2. Variance-covariance matrices for each of the regressions will be extracted to inform the parameter uncertainty estimates in the model and used in the probabilistic sensitivity analysis (PSA) as explained in section 3.2.4 below.

Table 2. Model parameters to inform long-term decision analytic model		
Transition probability	***Sources***	***Specific details of the fields in source***
Remaining in IT	NTP<br>NCRF	Time of withdrawal from IT<br>Time of withdrawal from IT
LTOT to IT	The trial	Proportion of people engaged in IT
IT to LTOT	NTP<br>NCRF	Time of withdrawal from IT<br>Time of withdrawal from IT
IT to SOLMP	NTP<br>NCRF	Time of completely stopping the use of opioids<br>Time of completely stopping the use of opioids
IT to D	PL	All-cause mortality data from ONS [39]
Remaining in LTOT	The trial	Number of people who do not engage in IT and remain in LTOT
SOLMP to LTOT	PtQ<br>GPRs	Medication data<br>Prescription data
LTOT to PT	PtQ<br>GPRs	Medication data<br>Prescription data
LTOT to D	PL	All-cause mortality data from ONS [39]
Remaining in PT	The trial<br>PtQ<br>GPRs	Number of people who remain in PT over time<br>Medication data<br>Prescription data
PT to LTOT	PtQ<br>GPRs	Medication data<br>Prescription data
PT to SOLMP	PtQ<br>GPRs	Medication data<br>Prescription data
PT to D	PL	All-cause mortality data from ONS [39]
Remaining in SOLMP	PtQ<br>GPRs	Medication data<br>Prescription data
SOMP to D		All-cause mortality rates from ONS [39]
Utility scores	***Source***	***Specific details of the source***
LTOT	PL<br>PtQ	Utility of opioid therapy [46]<br>Regression analysis of patient self-reported EQ-5D
PT	PtQ	Regression analysis of patient self-reported EQ-5D
IT	PtQ	Regression analysis of patient self-reported EQ-5D
SOLMP	PtQ	Regression analysis of patient self-reported EQ-5D

For peer review only - http://bmjopen.bmj.com/site/about/guidelines.xhtml

For peer review only

Costs	***Source***	***Specific details of the source***
LTOT	PL<br>PtQ<br>GPRs	Cost of opioid therapy per cycle [47]<br>Costs associated with self-reported resource use<br>Costs associated with resource use collected
PT	The trial<br>PtQ<br>GPRs	Cost associated with usual care<br>Costs associated with self-reported resource use<br>Costs associated with resource use collected
IT	The trial	Cost associated with intervention
SOLMP	PL	Assumption - 1x contact with GP [28]
Key for Table 2: D - Dead state, GPRs - GP records, IT - I-WOTCH tapering, LTOT - Long term opioid therapy, NCRF -nurse clinical record form, NTP - Nurse tapering plan, PL - Published literature, PT - Patient driven tapering, PtD - Patient diaries, PtQ - Patient questionnaire, SOLMP - Successful opioid-less management of pain, The trial - I-WOTCH [illegible]		

For peer review only

3.2.2 Incremental analysis

We will estimate the differential costs and QALYs predicted by the model and - where relevant - calculate the incremental cost-effectiveness ratio (ICER), defined as the ratio between the mean difference in costs (ΔC) and the mean difference in QALYs (ΔE). As decision makers need to assess whether an intervention is 'value for money' we will compare the ICER against two 'thresholds': one ranging between £20,000 and £30,000 per QALY gained to mimic the criterion used by NICE for its policy decisions, and another recently proposed by Lomas *et al* [40] who estimated this threshold to be between £5,000 and £15,000.

3.2.3 Subgroup analysis

In order to reflect variation between individuals in terms of their health benefit, resource use, and costs, we will run the Markov state transition model for a series of different patient profiles. This will enable us to reflect the impact that patient characteristics may have on the value for money assessment of I-WOTCH. The rationale for this approach stems from the recognition of the baseline risk (and possibly treatment effect) may vary between individuals based on their opioid use, pain severity, and pain conditions, for instance. The trial's pre-defined subgroups based on baseline variables are given below:

i) pain severity score (5 to 8, 9 to 11, and 12 to 15);
ii) opioid usage in MED (0 -20 mg, 30-59 mg, 60 -89 mg, 90-119 mg, 120 -149 mg, and >= 150 mg); and
iii) pain conditions (fibromyalgia, musculo-skeletal, arthritis, back pain, neurological, cancer, and other), and
iv) duration of opioid intake (Less than 1 year, 1-5 years, and more than 5 years).

3.2.4 Probabilistic sensitivity analyses

Sampling uncertainty in the model will be captured by characterising each model parameter with an appropriate probability distribution. We will derive the parameters to inform these distributions from the variance-covariance matrix obtained from each regression model.

For peer review only - http://bmjopen.bmj.com/site/about/guidelines.xhtml

Cholesky decomposition of each variance-covariance matrix will be used to make the simulation more efficient. Sampling uncertainty will be propagated through the model using Monte-Carlo simulation in the form of PSA to understand the effect on the predicted mean QALYs and mean costs associated with each treatment arm. These parameters will then be combined and compared against a range of feasible cost-effectiveness thresholds to obtain an estimate of the probability that I-WOTCH is cost-effective. This information will be represented graphically in the form of a cost-effectiveness acceptability curve (CEAC).[41]. We will also perform scenario analyses for the various distributional assumptions made, e.g. on the parametric survival distribution and compare the results for different assumptions.

Patient and public involvement

The Involvement of patient and public in the intervention design, development, and delivery are outlined in the I-WOTCH clinical protocol paper.[19] In brief, two lay advisers with chronic pain withdrawal of opioids and experience of clinical trial research have been recruited to the study. Additionally, prior to receiving funding for the study, meetings were held with volunteers with chronic malignant pain to receive input to the design of the intervention structure, duration of intervention and content to be covered. These meeting contributed also to the design of the study including randomisation, best usual care intervention, recruitment processes as well as outcome measures.

Ethics and dissemination

The study was approved by the Yorkshire & The Humber –South Yorkshire Research Ethics Committee (16/YH/0325). Appropriate local approvals were sought for each area in which recruitment was undertaken. The current protocol version is 1.7 date 31 July 2019 . The trial is registered with an International Standard Randomised Controlled Trial Number (ISRCTN) Register. ISRTC number 49470934 (6 Feb 2017).

DISCUSSION

The scale of the opioid crisis in the UK is not as severe as in the United States of America. However, it has been reported recently that opiates are a frequent cause of deaths due to drug poisoning in the UK.[42,43] To begin reviewing the benefits and risks of opioid medicine, and make recommendations for regulatory action, an expert working group has been formed by the Medicines and Healthcare Products Regulatory Agency (MHRA).[44] This protocol describes the first economic evaluation of an adjunct intervention (i.e. I-WOTCH) to support opioid tapering in patients with chronic non-malignant pain. Its findings will provide timely and significant results to inform policy recommendations on how best tackle the opioids epidemic in the UK and manage the complex landscape of opioids related health (and financial) risks.

A study that tested the effectiveness of an earlier version of I-WOTCH (i.e. the COPERS trial COping with persistent Pain, Effectiveness Research into Self-management) showed sustained benefits on depression and anxiety in people with musculoskeletal chronic pain.[45] The COPERS intervention was designed based on cognitive behavioural therapy principles. While in the COPERS trial more than 20% of participants used strong opioids this was not aimed at

For peer review only - http://bmjopen.bmj.com/site/about/guidelines.xhtml

opioid dose reduction. In contrast, the I-WOTCH intervention was designed to support opioid reduction and improve activities of daily living.

Strengths

This paper describes the first economic evaluation analysis of a complex intervention to support opioid tapering, which uses data collected alongside the first UK based RCT. A within-trial CCA will allow estimating relevant model parameters. The long-term impact on costs and health benefits from implementing the I-WOTCH programme in England will be assessed in a lifetime decision analytic model populated using trial and previously published data. This analysis is based on a robust conceptual model that reflects clinical practice and long-term adverse events associated with both opioid use and opioid withdrawal. This model will enable estimation of I-WOTCH's cost-effectiveness over a lifetime horizon and real-life subgroup analyses.

Limitations

Our decision problem and associated model structure are complex. I-WOTCH's effectiveness in the short and long term is associated to the occurrence / absence of several interlinked events that determine individual's transition from one health state to the other. I-WOTCH's 12-month trial follow up period is unlikely to allow to capture events of interest in the long-term. This may limit our ability to estimate all relevant transition probabilities on trial based IPD. A number of model parameters may need to be estimated from published sources and expert opinions. Data availability may limit the successful evaluation of the CEA model.

Acknowledgements

The authors would like to thank the members of the I-WOTCH trial team for their help in reviewing and acknowledge the inputs of the investigators contributing to the development of the trial.

Author contributions

SMK drafted the first version of this manuscript, CPIU is responsible for overseeing the design and implementation of the I-WOTCH economic analysis and for drafting subsequent versions of the manuscript, VSG contributed to the review of the literature of economic evaluation studies, AM provided expert advice and contributed to the draft of the manuscript. HS and SE are Co-Chief Investigators and oversee the running of the trial. MU has provided input into all aspects of the trial study design and support in running of the study. SMK, VSG, CPIU, HS, MU, SE and AM reviewed and approved the final version of the paper.

Funding statement

This project is funded by the National Institute of Health Research (NIHR), Health Technology Assessment (HTA) (project number 14/224/04). The views and opinions expressed therein are those of authors and do not necessarily reflect those of HTA, NIHR, or NHS.

For peer review only

Competing interests

MU was Chair of the NICE accreditation advisory committee until March 2017 for which he received a fee. He is chief investigator or co-investigator on multiple previous and current research grants from the UK National Institute for Health Research, Arthritis Research UK and is a co-investigator on grants funded by the Australian NHMRC. He is an NIHR Senior Investigator. He has received travel expenses for speaking at conferences from the professional organisations hosting the conferences. He is a director and shareholder of Clinvivo Ltd that provides electronic data collection for health services research. He is part of an academic partnership with Serco Ltd related to return to work initiatives. He is a co-investigator on a study receiving support in kind from Stryker Ltd. He has accepted honoraria for teaching/lecturing from consortium for advanced research training in Africa. He is an editor of the NIHR journal series, and a member of the NIHR Journal Editors Group, for which he receives a fee. SE is investigator on number of NIHR and industry sponsored studies. He received travel expenses for speaking at conferences from the professional organisations organising these conferences. SE attended advisory boards and provided consultancy service for Medtronic, Abbott, Boston Scientific and Mainstay Medical, none in relation to opioids. SE's department has received research funding from Medtronic. HS is director of Health Psychology Services Ltd, providing psychological services for a range of health related conditions. AM has received has received consultancy fees for participating in Pharmaceutical and Medical Device Industry Advisory Boards in the area of musculoskeletal pain. AM is member of the NICE Technology Appraisal Committee. CPIU is member of the NICE Medical Technologies Advisory Committee.

For peer review only

For peer review only - http://bmjopen.bmj.com/site/about/guidelines.xhtml

REFERENCES

1. Fayaz A, Croft P, Langford R, et al. Prevalence of chronic pain in the UK: a systematic review and meta-analysis of population studies. *BMJ open* 2016;6(6):e010364.
2. Curtis HJ, Croker R, Walker AJ, et al. Opioid prescribing trends and geographical variation in England, 1998–2018: a retrospective database study. *Lancet Psychiatry* 2019;6(2):140-50.
3. Taylor S, Annand F, Burkinshaw P, et al. Dependence and withdrawal associated with some prescribed medicines: an evidence review. *London: Public Health England* 2019. https://assets.publishing.service.gov.uk/government/uploads/system/uploads/attachment_data/file/829777/PHE_PMR_report.pdf (date accessed 16 December 2019).
4. Manchikanti L, Ailinani H, Koyyalagunta D, et al. A systematic review of randomized trials of long-term opioid management for chronic non-cancer pain. *Pain Physician* 2011;14(2):91-121.
5. Noble M, Treadwell JR, Tregear SJ, et al. Long-term opioid management for chronic noncancer pain. *Cochrane Database Syst Rev* 2010(1)
6. Els C, Jackson TD, Kunyk D, et al. Adverse events associated with medium-and long-term use of opioids for chronic non-cancer pain: an overview of Cochrane Reviews. *Cochrane Database Syst Rev* 2017(10)
7. Warner M, Trinidad JP, Bastian BA, et al. Drugs most frequently involved in drug overdose deaths: United States, 2010–2014. *Natl Vital Stat Rep* 2016
8. Moore RA, McQuay HJ. Prevalence of opioid adverse events in chronic non-malignant pain: systematic review of randomised trials of oral opioids. *Arthritis Res Ther* 2005;7(5):R1046.
9. Sullivan MD, Edlund MJ, Zhang L, et al. Association between mental health disorders, problem drug use, and regular prescription opioid use. *Arch Intern Med* 2006;166(19):2087-93.
10. Darcq E, Kieffer BL. Opioid receptors: drivers to addiction? *Nat Rev Neurosci* 2018:1.
11. Kim JY, Kim JH, Yee J, et al. Risk factors of opioid-induced adverse reactions in elderly male outpatients of Korea Veterans Hospital. *BMC Geriatr* 2018;18(1):293.
12. Bedson J, Chen Y, Ashworth J, et al. Risk of adverse events in patients prescribed long-term opioids: A cohort study in the UK Clinical Practice Research Datalink. *Eur J Pain* 2019
13. Dowell D, Haegerich TM, Chou R. CDC guideline for prescribing opioids for chronic pain—United States, 2016. *JAMA* 2016;315(15):1624-45.
14. Sandhu H, Underwood M, Furlan A, et al. What interventions are effective to taper opioids in patients with chronic pain? *BMJ* 2018;362:k2990.
15. Sullivan MD, Turner JA, DiLodovico C, et al. Prescription opioid taper support for outpatients with chronic pain: a randomized controlled trial. *J Pain* 2017;18(3):308-18.
16. Gowing L, Ali R, White JM. Buprenorphine for the management of opioid withdrawal. *Cochrane Database Syst Rev* 2009(3)
17. Frank JW, Lovejoy TI, Becker WC, et al. Patient outcomes in dose reduction or discontinuation of long-term opioid therapy: a systematic review. *Ann Intern Med* 2017;167(3):181-91.
18. Gowing L, Ali R, White JM. Opioid antagonists with minimal sedation for opioid withdrawal. *Cochrane Database Syst Rev* 2017(5)

For peer review only - http://bmjopen.bmj.com/site/about/guidelines.xhtml

19. Sandhu HK, Abraham C, Alleyne S, et al. Testing a support programme for opioid reduction for people with chronic non-malignant pain: the I-WOTCH randomised controlled trial protocol. *BMJ Open* 2019;9(8):e028937.
20. National Institute for Health and Clinical Excellence. Guide to the methods of technology appraisal 2013 [Available from: https://www.nice.org.uk/process/pmg9/resources/guide-to-the-methods-of-technology-appraisal-2013-pdf-2007975843781 (date accessed 16 December 2019)].
21. A brief guide to the PROMIS© Pain Interference instruments [Available from: http://www.healthmeasures.net/images/PROMIS/manuals/PROMIS_Pain_Interference_Scoring_Manual.pdf(date (date accessed 16 December 2019)].
22. Vernon MK, Reinders S, Mannix S, et al. Psychometric evaluation of the 10-item Short Opiate Withdrawal Scale-Gossop (SOWS-Gossop) in patients undergoing opioid detoxification. *Addict Behav* 2016;60:109-16.
23. Herdman M, Gudex C, Lloyd A, et al. Development and preliminary testing of the new five-level version of EQ-5D (EQ-5D-5L). *Qual Life Res* 2011;20(10):1727-36.
24. Position statement on use of the EQ-5D-5L value set for England [Available from: https://www.nice.org.uk/about/what-we-do/our-programmes/nice-guidance/technology-appraisal-guidance/eq-5d-5l (date accessed 16 December 2019)].
25. Devlin NJ, Shah KK, Feng Y, et al. Valuing health-related quality of life: An EQ-5 D-5 L value set for England. *Health Econ* 2018;27(1):7-22.
26. van Hout B, Janssen MF, Feng YS, et al. Interim scoring for the EQ-5D-5L: mapping the EQ-5D-5L to EQ-5D-3L value sets. *Value Health* 2012;15(5):708-15.
27. Department of Health. National Schedule of Reference Costs 2017–2018 2018 [Available from: https://improvement.nhs.uk/resources/reference-costs/ (date accessed 16 December 2019)].
28. Curtis L, Burns A. Unit Costs of Health and Social Care 2018 [Available from: https://www.pssru.ac.uk/project-pages/unit-costs/unit-costs-2018/ (date accessed 16 December 2019)].
29. Curtis L. Unit costs of health and social care 2013 [Available from: http://www.pssru.ac.uk/project-pages/unit-costs/2013/ (date accessed 16 December 2019)].
30. Curtis L, Burns A. Unit Costs of Health and Social Care 2017 [Available from: https://www.pssru.ac.uk/project-pages/unit-costs/unit-costs-2017/ (date accessed 16 December 2019)].
31. Inflation and price indices 2019 [Available from: https://www.ons.gov.uk/economy/inflationandpriceindices (date accessed 16 December 2019)].
32. Joint Formulary Committee. British national formulary, 76 Edition 2019 [Available from: http://www.bnf.org/bnf/index.htm (date accessed 05 June 2019)].
33. R : A Language and Environment for Statistical Computing [program], 2014.
34. Faria R, Gomes M, Epstein D, et al. A guide to handling missing data in cost-effectiveness analysis conducted within randomised controlled trials. *Pharmacoeconomics* 2014;32(12):1157-70.
35. Wailoo AJ, Hernandez-Alava M, Manca A, et al. Mapping to Estimate Health-State Utility from Non-Preference-Based Outcome Measures: An ISPOR Good Practices for Outcomes Research Task Force Report. *Value Health* 2017;20(1):18-27.

For peer review only - http://bmjopen.bmj.com/site/about/guidelines.xhtml

36. Hernandez M, Wailoo A, Pudney S, et al. Modelling generic preference based outcome measures - development and comparison of methods. *Health Technol Assess* 2019
37. Mihaylova B, Briggs A, O'Hagan A, et al. Review of statistical methods for analysing healthcare resources and costs. *Health Econ* 2011;20(8):897-916.
38. Lin D. Linear regression analysis of censored medical costs. *Biostatistics* 2000;1(1):35-47.
39. Office for National Statistics. Deaths registered in England and Wales [Available from: https://www.ons.gov.uk/peoplepopulationandcommunity/birthsdeathsandmarriages/deaths (date accessed 17 December 2019)].
40. Lomas J, Martin S, Claxton K. Estimating the Marginal Productivity of the English National Health Service From 2003 to 2012. *Value Health* 2019;22(9):995-1002.
41. Fenwick E, O'Brien BJ, Briggs A. Cost-effectiveness acceptability curves–facts, fallacies and frequently asked questions. *Health Econ* 2004;13(5):405-15.
42. John E, Butt A, McQuade G. Deaths related to drug poisoning in England and Wales: 2018 registrations. 2019. https://www.ons.gov.uk/peoplepopulationandcommunity/birthsdeathsandmarriages/deaths/bulletins/deathsrelatedtodrugpoisoninginenglandandwales/2018registrations#links-to-related-ons-information (date accessed 21 January 2020).
43. Taylor S, Annand F, Burkinshaw P, et al. Dependence and withdrawal associated with some prescribed medicines [Available from: https://assets.publishing.service.gov.uk/government/uploads/system/uploads/attachment_data/file/829777/PHE_PMR_report.pdf (date accessed 16 December 2019)].
44. Opioid Expert Working Group meets at MHRA [Available from: https://www.gov.uk/government/news/opioid-expert-working-group-meets-at-mhra (date acessed 12 December 2019)].
45. Taylor SJ, Carnes D, Homer K, et al. Novel Three-Day, Community-Based, Nonpharmacological Group Intervention for Chronic Musculoskeletal Pain (COPERS): A Randomised Clinical Trial. *PLoS Med* 2016;13(6):e1002040.
46. Ikenberg R, Hertel N, Andrew Moore R, et al. Cost-effectiveness of tapentadol prolonged release compared with oxycodone controlled release in the UK in patients with severe non-malignant chronic pain who failed 1st line treatment with morphine. *J Med Econ* 2012;15(4):724-36.
47. Sullivan W, Hirst M, Beard S, et al. Economic evaluation in chronic pain: a systematic review and de novo flexible economic model. *Eur J Health Econ* 2016;17(6):755-70.

For peer review only

LIST OF ABBREVIATIONS

ADL - Activities of Daily Living

BNF - British National Formulary

CCA - Cost-Consequences Analysis

CEA - Cost-Effectiveness Analysis

CEAC - Cost-Effectiveness Acceptability Curve

COPERS - COping with persistent Pain, Effectiveness Research into Self-management

D - Death

GLM - Generlised Linear Models

GP - General Practitioner

HRG - Healthcare Resource Group

HTA – Health Technology Assessment

ICER - Incremental Cost-Effectiveness Ratio

IPD – Individual Patient Data

IPW - Inverse Probability Weighting

ISRCTN - International Standard Randomised Controlled Trial Number

ITT - Intention To Treat

IT - I-WOTCH Tapering

I-WOTCH - Improving the Wellbeing of people with Opioid Treated CHronic Pain

LTOT - Long Term Opioid Therapy

MHRA - Medicines and Healthcare products Regulatory Agency

NHS - National Health Service

NICE - National Institute for Health and Care Excellence

NIHR - National Institute of Health Research

MED - Morphine Equivalent Dose

For peer review only

For peer review only - http://bmjopen.bmj.com/site/about/guidelines.xhtml

ONS - Office of National Statistics

PROMIS-PI-SF-8A - Patient Reported Outcomes Measurement Information System Pain Intensity Short Form-8A

PSA - Probabilistic Sensitivity Analysis

PSAE - Persistent / Serious Adverse Events

PSSRU - Personal and Social Services Research Unit

PT - Patient-driven Tapering

QALY - Quality Adjusted Life Year

RCT - Randomised Controlled Trial

SD - Standard Deviation

ShOWS - Short Opiate Withdrawal Scale

SOLMP - Successful Opioid-Less Management of Pain

TEAE - Transient/Emergent Adverse Events

For peer review only

For peer review only - http://bmjopen.bmj.com/site/about/guidelines.xhtml

FIGURE CAPTIONS

Figure 1: Simplified model structure showing patient flow during I-WOTCH trial. At any time, Individuals will be allocated to any of the five health states (shown as ovals). Individuals start in the long-term opioid therapy (LTOT) health state. Depending on the trial arm, they will move to either I-WOTCH tapering (IT) or patient driven tapering (PT). The transition from LTOT to IT occurs only at the start of the trial and patients cannot go back IT once they withdraw from IT tapering. The patients who have tapered their opioid doses completely will be in the health state successful opioid less management of pain (SOLMP). The arrows 'spearheads indicate the direction of allowed transition from one state to the other. Dead (D) state is an absorbing state where no transitions from the dead state are allowed.

For peer review only

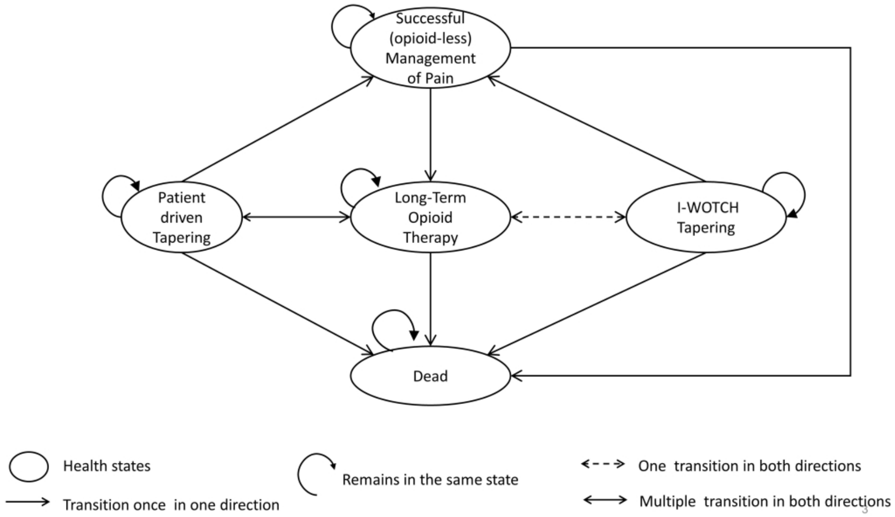

Figure 1: Simplified model structure showing patient flow during I-WOTCH trial. At any time, Individuals will be allocated to any of the five health states (shown as ovals). Individuals start in the long-term opioid therapy (LTOT) health state. Depending on the trial arm, they will move to either I-WOTCH tapering (IT) or patient driven tapering (PT). The transition from LTOT to IT occurs only at the start of the trial and patients cannot go back IT once they withdraw from IT tapering. The patients who have tapered their opioid doses completely will be in the health state successful opioid less management of pain (SOLMP). The arrows 'spearheads indicate the direction of allowed transition from one state to the other. Dead (D) state is an absorbing state where no transitions from the dead state are allowed.

338x190mm (300 x 300 DPI)

Supplementary Material

Resource use and costing for the I-WOTCH Intervention

Table A1. Resource use and costs related to running I-WOTCH intervention		
**I-WOTCH intervention running costs**		
Category	***Resource use***	***Unit cost***
Number of participants	Number of participants enrolled in the course	
Facilitator costs	Total number and duration of sessions per facilitator	Fixed fee per session (pension and national Insurance contributions included). Alternatively, grade (if NHS) or salary
Facilitator travel costs	Total number and type (i.e. car, train, etc.) of journeys	Train or bus fare and fixed fee per mile.
Administrator costs	Number of days / months dedicated to coordinate implementation of I-WOTCH intervention	Salary used to estimate fixed daily rate (pension and national insurance contributions included)
Facility costs	Number of days facility has been hired per course delivered	Daily rate for venue
Hospitality costs	Food and drink	
Consumables	Stationary (e.g. letters, stamps, notebooks, pens, pencils, etc.)	
Course materials costs		
My Opioid Manager Manual	Total number of manuals produced and delivered	Total printing costs and copy right fee
Relaxation Package	Total number of CDs produced and delivered	Total cost of CDs

For peer review only

Table A2. Resource use and costs related to training related to I-WOTCH intervention		
**Training costs**		
Category	***Resource use***	***Unit cost***
Trainer costs	Number of days / months - per trainer - dedicated to train facilitators to deliver I-WOTCH intervention	Fixed daily rate
Trainer travel cost	Total number and type (i.e. car, train, etc.) of journeys	Train or bus fare and fixed fee per mile
Facilitator costs	Total number and duration of sessions per facilitator	Fixed fee per session (pension and national insurance contributions included) or salary
Facilitator travel costs	Total number and type (i.e. car, train, etc.) of journeys	Train or bus fee and fixed fee per mile
Administrator costs	Number of days / months dedicated to coordinate implementation of I-WOTCH intervention	Salary used to estimate fixed daily rate (pension and national insurance contributions included)
Facility costs	Number of days facility has been hired per course delivered	Daily rate for venue
Hospitality costs		
Consumables		
Course materials costs		
My Opioid Manager Manual	Total number of manuals produced and delivered	Total printing costs
Relaxation Package	Total number of CDs produced and delivered	Total cost of CDs

For peer review only

Table A3. Data collection form used to characterise the I-WOTCH intervention facilitators	
**Characteristics of course facilitators**	
Variable	***Description***
Facilitator ID	Define unique key
Site	
Age	In years
Gender	
Ethnicity	Same categories used for participants
Type	Healthcare provider<br>Lay person
Profession / Experience	
Years of professional experience (for health care professional (HCP)) or facilitation (lay person)	
Number of curses facilitated	

Table A4. Data collection form used to characterise the course in I-WOTCH intervention	
**Course venues, facilitators, and participants**	
Variable	***Description***
Course ID	
Venue	
Facilitator	Facilitator profession/experience
Number enrolled	
Attendance	Attendance per patient per session

For peer review only

BMJ Open

Protocol for an economic analysis of the randomised controlled trial of improving the wellbeing of people with opioid treated chronic pain: I-WOTCH study

Journal:	*BMJ Open*
Manuscript ID	bmjopen-2020-037243.R1
Article Type:	Protocol
Date Submitted by the Author:	16-Jun-2020
Complete List of Authors:	Manchira Krishnan, Sheeja; University of York, Department of Health Sciences<br>Gc, Vijay; University of York Centre for Health Economics, ; University of East Anglia Norwich Medical School,<br>Sandhu, Harbinder; University of Warwick Warwick Medical School, Clinical Trials Unit<br>Underwood, Martin; University of Warwick, Warwick Clinical Trials Unit, Warwick Medical School; University Hospitals Coventry and Warwickshire NHS Trust<br>Eldabe, Sam; James Cook University Hospital, Pain Department<br>Manca, Andrea; University of York, Center for Health Economics<br>Iglesias, C; University of York, Department of Health Sciences
**Primary Subject Heading**:	Health economics
Secondary Subject Heading:	Public health
Keywords:	HEALTH ECONOMICS, PAIN MANAGEMENT, Protocols & guidelines < HEALTH SERVICES ADMINISTRATION & MANAGEMENT

SCHOLARONE™
Manuscripts

BMJ

I, the Submitting Author has the right to grant and does grant on behalf of all authors of the Work (as defined in the below author licence), an exclusive licence and/or a non-exclusive licence for contributions from authors who are: i) UK Crown employees; ii) where BMJ has agreed a CC-BY licence shall apply, and/or iii) in accordance with the terms applicable for US Federal Government officers or employees acting as part of their official duties; on a worldwide, perpetual, irrevocable, royalty-free basis to BMJ Publishing Group Ltd ("BMJ") its licensees and where the relevant Journal is co-owned by BMJ to the co-owners of the Journal, to publish the Work in this journal and any other BMJ products and to exploit all rights, as set out in our licence.

The Submitting Author accepts and understands that any supply made under these terms is made by BMJ to the Submitting Author unless you are acting as an employee on behalf of your employer or a postgraduate student of an affiliated institution which is paying any applicable article publishing charge ("APC") for Open Access articles. Where the Submitting Author wishes to make the Work available on an Open Access basis (and intends to pay the relevant APC), the terms of reuse of such Open Access shall be governed by a Creative Commons licence – details of these licences and which Creative Commons licence will apply to this Work are set out in our licence referred to above.

Other than as permitted in any relevant BMJ Author's Self Archiving Policies, I confirm this Work has not been accepted for publication elsewhere, is not being considered for publication elsewhere and does not duplicate material already published. I confirm all authors consent to publication of this Work and authorise the granting of this licence.

For peer review only

Protocol for an economic analysis of the randomised controlled trial of improving the wellbeing of people with opioid treated chronic pain: I-WOTCH study

Sheeja Manchira Krishnan[1], Vijay S. Gc[2], Harbinder K. Sandhu[3], Martin Underwood[3,4], Sam Eldabe[5], Andrea Manca[2], and Cynthia P Iglesias Urrutia[1] on behalf of the I-WOTCH team

[1] Department of Health Sciences, University of York, Heslington, York, YO10 5DD, UK

[2] Centre for Health Economics, University of York, Heslington, York, YO10 5DD, UK

[3] Warwick Clinical Trials Unit, Warwick Medical School, University of Warwick, Gibbet Hill Campus, Coventry, CV4 7AL, UK

[4] University Hospitals of Coventry and Warwickshire, Clifford Bridge Road, Coventry CV2 2DX, UK

[5] Pain Department, The James Cook University Hospital, Marton Road, Middlesbrough, TS4 3BW, UK

Correspondence:

Dr. Cynthia P Iglesias Urrutia

Department of Health Sciences

University of York

Heslington

York YO10 5DD, UK

Email: cynthia.iglesias@york.ac.uk

Phone: 01904 32 1820

For peer review only

Keywords: Health economics analysis plan, within-trial analysis, model-based analysis, cost-effectiveness analysis, opioid tapering

Word count: 3892 (excluding title page, abstract, acknowledgements, funding statement, author contributions, and references)

Number of tables and figures: Figures 1, Tables 2

For peer review only - http://bmjopen.bmj.com/site/about/guidelines.xhtml

ABSTRACT

Introduction: Over the last two decades, the use of opioids for the treatment of chronic pain in England has steadily increased despite lack of evidence of both long-term effectiveness in pain relief and significant, well documented physical and mental adverse events. Guidelines recommend tapering when harms outweigh benefits, but the addictive nature of opioids hinders simple dose reduction strategies. Improving the Wellbeing of people with Opioid Treated CHronic pain (I-WOTCH) trial tests a multicomponent self-management intervention aimed to help patients with chronic non-malignant pain taper opioid doses. This paper outlines the methods to be used for the economic analysis of the I-WOTCH intervention compared to the best usual care.

Methods and analysis: Economic evaluation alongside the I-WOTCH study, prospectively designed to identify, measure, and value key healthcare resource use and outcomes arising from the treatment strategies being compared. A within-trial cost-consequences analysis and a model-based long-term cost-effectiveness analysis will be conducted from the National Health Service and Personal Social Service perspective in England. The former will quantify key parameters to populate a Markov model designed to estimate the long-term cost and quality-adjusted life years of the I-WOTCH programme against best usual care. Regression equations will be used to estimate parameters such as transition probabilities, utilities, and costs associated with the model's states and events. Probabilistic sensitivity analysis will be used to assess the impact of parameter uncertainty onto the predicted costs and health outcomes, and the resulting value for money assessment of the I-WOTCH programme.

Ethics and dissemination: Full ethics approval was granted by Yorkshire & The Humber – South Yorkshire Research Ethics Committee on September 13th, 2016 (16/YH/0325). Current protocol: version 1.7, date 31 July 2019. Findings will be disseminated in peer-reviewed journals, scientific conferences, newsletters, and websites.

Registration details: International Standard Randomised Controlled Trial Number: 49470934 (6 Feb 2017).

Word count: 300 words

For peer review only

Article summary

Strengths and limitation of this study:

- First economic evaluation of a complex intervention (I-WOTCH) to support opioid tapering;
- The economic analyses use patient-level information to inform a de-novo decision model;
- I-WOTCH's decision model will enable estimation of the costs, health consequences, and uncertainty associated with opioid use and tapering over a lifetime horizon;
- Valuable evidence for potential implementation of the self-management support program aimed at opioid tapering;
- Uncertainties may remain as to the long-term effectiveness and cost-effectiveness of the I-WOTCH intervention due to the limited follow-up duration of the current trial.

For peer review only

1. Background

Nearly half of the UK adult population (43%) is living with chronic pain (i.e. pain lasting >3 months), the prevalence of which increases with age.[1] Opioids are commonly prescribed for chronic pain and a recent study reports an increase in the number of opioid prescriptions (approximately 34%) in England between 1998 and 2016.[2] Analysis of prescription data shows that after this long increasing trend, there is a slight decrease in the annual number of opioid prescriptions for pain since 2016.[3] Despite the demonstrated short-term effectiveness of opioids, evidence of their long-term impact in terms of pain relief and improvement in functional status is scant.[4,5] This situation is compounded by concerns over the fact that long-term use of opioids can lead to adverse events (at an estimated absolute rate of 78% in trials using a placebo as a comparator) affecting the respiratory, cardiovascular, gastrointestinal, and central nervous systems.[6] These include common adverse events such as dry mouth, nausea, and constipation as well as more serious adverse events including sleep-disordered breathing, respiratory depression, and opioid-related deaths (at an absolute rate of 7.5%).[6-8] Equally concerning is the fact that opioids use is associated with mental health and anxiety disorders, major depression, and dysthymia.[9] Their long-term use may cause problematic patterns of substances use, leading to substance use disorders (i.e. abuse and dependence).[10] Risk factors for opioid-related adverse events include older age, higher doses of this class of drugs and their long-term use.[11,12]

Clinical guidelines for prescribing opioids in chronic pain recommend tapering when the possible harms from their use outweigh any expected benefits [13] and yet, to our knowledge, no validated protocol or intervention exist to help patients reduce their opioid doses and manage their chronic pain.[14] A number of studies have evaluated interventions (e.g. acupuncture, ketamine assisted dose reduction, and behavioural strategies such as motivational interviewing, psychiatric consultation, cognitive behavioural therapy, and mindfulness) that support opioid tapering.[15-18] The conclusions drawn by these studies are limited by the quality of their design or insufficient follow-up period.[14] Relevant for our study, there are no existing economic evaluations of opioid tapering strategies in the management of chronic non-malignant pain.

Improving the Well-being of People with Opioid Treated Chronic Pain (I-WOTCH) is an NIHR funded randomised controlled trial (RCT) evaluating the effectiveness and cost-effectiveness of a patient-centred, multicomponent self-management intervention targeting withdrawal of strong opioids among those living with chronic non-malignant pain.[19] The I-WOTCH trial is designed to help people reduce their opioid consumption, manage pain interference, and enhance their quality of life.

This paper describes the protocol for the economic analysis that has been designed as an integral part of the I-WOTCH trial.

2. Methods

2.1 Study details (population, setting, location, intervention, and comparator)

For peer review only - http://bmjopen.bmj.com/site/about/guidelines.xhtml

A detailed study protocol for the I-WOTCH trial has been reported in a separate manuscript.[19] Briefly, I-WOTCH is a multi-site, patient-centred, open randomised controlled trial enrolling adult patients with non-malignant chronic pain in England. The trial's target sample size is 542 participants, individually randomised (1:1) to receive the I-WOTCH intervention or best usual care. The I-WOTCH intervention is an 8-10 weeks course - consisting of a mixture of group sessions led by two trained I-WOTCH facilitators, two one-to-one individual sessions and two telephone consultations with the I-WOTCH trained nurse facilitator - adjunct to best usual care. Best usual care consists of general practitioner (GP) care with relaxation package and a booklet called 'My Opioid Manager'.

2.2 Planned start and end dates

The I-WOTCH study started in September 2016 and is expected to end in March 2021. The economic analysis is expected to begin in October 2020.

2.3 Type of economic evaluation (cost-consequences and cost-effectiveness analyses)

Two types of economic analysis will be conducted: a within-trial cost-consequences analysis (CCA) and a long-term model-based cost-effectiveness analysis (CEA), the details of these are provided in section 3.

2.4 Study perspective

The perspective for both the CCA and CEA will be that of the National Health Service (NHS) and Personal Social Service for England.

2.5 Time horizon

The CCA and CEA will adopt a 12-month and a patients' life time horizons, respectively.

2.6 Discount rate

Estimates of mean cost and health benefits observed during the 12-month trial follow-up period will not be discounted (for the purpose of the CCA), while those predicted to accrue beyond the study follow-up (for the purpose of the CEA) will be discounted using a 3.5% annual discount rate as per National Institute for Health and Care Excellence (NICE) guidelines.[20]

2.7 Identification, measurements, and valuation of health outcomes

Table 1 describes the variables of interest to the economic analyses of I-WOTCH, the frequency of data collection, and data sources.

Table 1 Data collection strategy for I-WOTCH's economic analyses

Data collected	Source	Time of collection
Baseline data		
Age	PtQ	Baseline
Gender	PtQ	Baseline

For peer review only - http://bmjopen.bmj.com/site/about/guidelines.xhtml

Ethnic group	PtQ	Baseline
Current work status	PtQ	Baseline
Age at leaving full time education	PtQ	Baseline
Pain duration	PtQ	Baseline
Opioid intake duration	PtQ	Baseline
Pain conditions	PtQ<br>GPRs	Baseline<br>At 12 months only
Pain severity stratification group	PtQ	Baseline
Measures of health benefit		
Activities of Daily Living (PROMIS PI-SF-8A)	PtQ	Baseline, 4, 8 and 12 months
Severity of opioid withdrawal symptoms (ShOWS)	PtQ<br>PtD	Baseline, 4, 8 and 12 months<br>Weekly over first 4 months
Generic health related quality of life (EQ-5D-5L)	PtQ<br>PtD	Baseline, 4, 8 and 12 months<br>Weekly over first 4 months
Resource use (volume, admissions, consultations, attendances and/or contacts)		
Medications	PtQ<br>GPRs	Baseline, 4, 8 and 12 months<br>At 12 months only
Hospital	PtQ<br>GPRs	Baseline, 4, 8 and 12 months<br>At 12 months only
Hospital outpatient	PtQ<br>GPRs	Baseline, 4, 8 and 12 months<br>At 12 months only
GP surgery	PtQ<br>GPRs	Baseline, 4, 8 and 12 months<br>At 12 months only
GP home	PtQ<br>GPRs	Baseline, 4, 8 and 12 months<br>At 12 months only
GP telephone	GPRs	At 12 months only
Practice nurse	PtQ<br>GPRs	Baseline, 4, 8 and 12 months<br>At 12 months only
Practise nurse telephone	GPRs	At 12 months only
District nurse (i.e. at home)	PtQ<br>GPRs	Baseline, 4, 8 and 12 months<br>At 12 months only
NHS 111	GPRs	At 12 months only
Occupational therapist	PtQ	Baseline, 4, 8 and 12 months
Counsellor	PtQ	Baseline, 4, 8 and 12 months
Psychologist	PtQ	Baseline, 4, 8 and 12 months
Social worker	PtQ	Baseline, 4, 8 and 12 months
Physiotherapist	PtQ	Baseline, 4, 8 and 12 months
Referrals	GPRs	At 12 months only
Investigations	GPRs	At 12 months only
Ambulance incidents	GPRs	At 12 months only
Accident and emergency	GPRs	At 12 months only
Other	PtQ<br>GPRs	Baseline, 4, 8 and 12 months<br>At 12 months only
Key for Table 1: GPRs - GP records, PtD - Patient diaries, and PtQ - Patient questionnaire		

For peer review only - http://bmjopen.bmj.com/site/about/guidelines.xhtml

The CCA will consider the following three health outcome measures over the 12-month trial follow-up period:

1) *Activities of Daily Living* (ADL) - measured using the patient reported outcomes measurement information system pain intensity short form-8A questionnaire (PROMIS-PI-SF-8A);[21]
2) *Severity of opioid withdrawal symptoms* - measured using the short opiate withdrawal scale (ShOWS);[22]
3) The *5-level EQ-5D* version (EQ-5D-5L) instrument - a preference-based generic measure of health related quality of life typically used in healthcare economic evaluation.[23,24]

The PROMIS-PI-SF-8A comprises eight questions rated on a scale of 1-5 that aim to measure the degree of interference of pain on day-to-day activities, work around the home, ability to participate in social activities, household chores, fun activities, enjoyment of social activities, enjoyment of life, and family life. The total raw score is the sum of individual responses to each question thus forming a maximum score of forty and a minimum score of eight. Higher scores reflect large interference or change in participant's ability to perform daily activities. This instrument supports calculation of a common metric and will be converted to a T score.[21]

ShOWS identifies the severity of opiate withdrawal symptoms on ten different categories namely: feeling sick, stomach cramps, muscle spasms, feeling of coldness, heart pounding, muscular tension, aches and pains, yawning, runny eyes, and insomnia. The response to each question is attributed to an individual score of 0 to 3, with 0 indicating no symptoms, 1 mild, 2 moderate, and 3 severe.[22] Higher overall scores (estimated as the sum of all individual scores with a maximum score of thirty and a minimum score of zero) indicate higher severity of opioid withdrawal symptoms.

The EQ-5D-5L questionnaire [23] describes health in five domains (mobility, self-care, usual activities, pain or discomfort, and anxiety or depression). Each domain has five levels of severity (1 - no problems/2 - slight problems/3 - moderate problems/4 - severe problems/5 - unable to do). A response consists of a sequence of five digits, e.g. 12315, 12112, which represent the level of severity on each domain reported by the respondent. Combinations of the levels of the five domains describe 3125 possible health states. Several valuation studies have been carried out in the literature to estimate value sets for a given country/region. These studies used methods consistent with economic theory to elicit the respondent's preferences towards the health states defined by the EQ-5D. A value set to calculate utility values for the EQ-5D-5L have been published recently [25] but is still subject to methodological controversy. Until the controversies are resolved, we will convert EQ-5D-5L responses onto the EQ-5D-3L scale using the mapping function developed by van Hout *et al* [26] following current NICE's recommendation.[24]

The CEA will integrate predicted survival and EQ-5D index scores (for each model state and clinical event) to derive an estimate of quality-adjusted life years (QALYs) - the health benefit of choice in our CEA - under the I-WOTCH and best usual care strategies.

For peer review only - http://bmjopen.bmj.com/site/about/guidelines.xhtml

2.8 Identification, measurement and valuation of resource use

As described in Table 1, patient-level resource use data are collected in the trial and will be complemented by information routinely collected in GP records. Costing will be carried out in UK pound sterling at 2019 prices. Unit cost for tests, investigations, inpatient hospital admissions and day care procedures will be estimated using National Reference Costs and Healthcare Resource Group (HRG) codes.[27] Referrals and consultations will be costed using Personal and Social Services Research Unit (PSSRU) statistics.[28] If necessary, we will use unit costs from previous versions of the PSSRU report [29,30] and inflate them to the year 2019 using inflation and price indices from the Office of National Statistics (ONS).[31] Costs associated with usual care will include the cost of the relaxation CD (i.e. printing and production costs) and 'My Opioid Manager'.

2.8.1 Microcosting of I-WOTCH intervention

A microcosting of the resources required to provide the I-WOTCH intervention will be conducted. A detailed description of the categories of resource use to be considered, their associated unit costs and the data collection forms used in the I-WOTCH microcosting exercise is provided in the Supplementary Material (Annex A Tables A1 - A4). Salary, facility, and travel costs will be assumed to be independent of the number of participants. Salaries for the facilitators and trainers will be estimated based on average daily salary by grade. The facility costs will be calculated based on the number of venues hired, number of days hired, and daily venue hire rate. Travel costs will be considered as fixed cost per mile. Unit costs for nurses' time for face-to-face and telephone consultations will be extracted from the PSSRU 2019.[28] A per participant, locality, and course cost of I-WOTCH intervention will be reported.

2.8.2 Cost of medication

Unit costs of the medications (Table 1) will be obtained from the British National Formulary (BNF).[32] For each strength and preparation of a given pain killer drug, we will extract the cost per pack (or bottle) from the BNF and we will calculate the relevant morphine equivalent dose (MED) using the same algorithm used in the I-WOTCH clinical analysis. This is being updated from that used to estimate MED for stratification at the time of randomisation. For each opioid-based medication reported to have been used by individuals in the I-WOTCH study we will estimate their unit cost per MED and use it to estimate a weighted average cost per MED over all opioid medications used in the trial.

2.9 Modelling

A Markov state-transition model has developed to facilitate the estimation of the long-term mean costs and QALYs associated with the I-WOTCH intervention and usual care. The initial conceptual structure of the model was informed by a systematic analysis of the components of the I-WOTCH intervention. This task was supported and enhanced by critically appraising published decision models that evaluated the use of opioids in chronic non-malignant pain. The face validity of the model structure was further refined by holding a series of meetings with the project team involving clinical experts from the I-WOTCH study. This process is described in detail in a separate related manuscript currently in preparation.

For peer review only - http://bmjopen.bmj.com/site/about/guidelines.xhtml

Figure 1 depicts a schematic representation of our final model structure, which is organised around five key states (i.e. represented as ovals in Figure 1). At any time period, patients can be in one of the following mutually exclusive states:

a) long-term opioid therapy (LTOT) - representing individuals candidate for the intervention, who have been using strong opioids for more than 3 months;
b) I-WOTCH tapering (IT) - individuals committed to tapering as part of the I-WOTCH intervention;
c) patient driven tapering (PT) - individuals self-committed to tapering without the intervention;
d) successful opioid-less management of pain (SOLMP) - individuals completed withdrawal from opioid use; and
e) dead (D).

Each state is associated with an average cost and utility score, derived using the methods described in section 3.1.2. Spearheads of arrows in Figure 1 represent the directions of allowed transitions per cycle. LTOT is the starting state for all patients in the I-WOTCH trial. Individuals in LTOT can engage with IT only once but are able to initiate PT at their will. Whilst they remain in the LTOT state, individuals can experience opioid related transient/emergent adverse events (TEAE) or persistent/serious adverse events (PSAE). Occasionally, adverse events may trigger a transition from LTOT to PT. Individuals in IT or PT may withdraw from opioid tapering at any point in time and go back to LTOT. Individuals in IT or PT can transit to SOLMP state only when they are no longer taking any opioids. Finally, individuals are at risk of death at any point in time (i.e. transitions into the absorbing dead state are allowed from the LTOT, IT, PT, and SOLMP states). A paper describing a detailed description of the conceptualization of the model, health states definition, clinical events, and associated transitions is in preparation. To maximise the use of the data collected during the trial and to model the events and transitions observed during the treatment and follow-up period, the state transition model uses a weekly cycle length during the first 4 months and monthly cycles beyond that.

3. Analysis

Statistical analysis for the CCA and CEA will be conducted using I-WOTCH's individual patient data (IPD) collected on resource use and outcomes, analysed on an intention to treat (ITT) basis. Statistical analysis and decision models will be implemented in R.[33]

3.1 Within-trial cost-consequences analysis

The CCA will focus on estimating and reporting mean health outcomes, resource use, and costs by treatment arm at each follow up interval. No incremental analysis will be conducted. Total costs will be the sum of the costs of the healthcare resource items (described in Table 1) that patients used during the study period, plus the cost of either the I-WOTCH intervention or the usual care. Mean health outcomes will include those described in section 2.7 and reported at each follow-up period by treatment arm.

3.1.1 Handling missing data

For peer review only - http://bmjopen.bmj.com/site/about/guidelines.xhtml

We will assess the extent of missing data in the patient-level costs and health outcomes collected during I-WOTCH's 12-month follow-up and apply appropriate methods to handle the issue following the recommendations by Faria *et al.*[34]

3.1.2 Statistical analysis

Descriptive statistics (i.e. mean, standard deviation (SD), lower and upper quartiles, minimum and maximum values) for all continuous variables reported in Table 1 plus total costs will be estimated by trial arm. Histograms and/or box-plots will be used to represent these variables graphically. Binary and categorical variables will be represented in terms of percentages. The impact of patient's baseline characteristics (i.e. age, gender, pain severity, opioid use, duration of opioid intake, and opioid medication related pain condition) as predictors of outcomes will be explored in a series of regression models fitted to ADL, ShOWS, EQ-5D-3L utility scores, and cost data to inform the estimation of the Markov model's input parameters.

We will map EQ-5D-5L utility scores into EQ-5D-3L utility scores.[24,26] To account for idiosyncrasies of the EQ-5D-3L (i.e. multimodality, truncated support, and left-skewness) we will use a series of regression models (including mixture of beta models, adjusted limited dependent variable mixture models, two-part models). Each model goodness of fit and assessment of their predictive ability will be carried out using the methods recommended by Hernandez-Alava *et al*.[35,36]

Many methods have been used to analyse cost data.[37] We will use generalised linear models (GLM) (with Gaussian or gamma distributed errors and identity or log links) and where necessary, two-part extensions of these models to account for any mass at zero, the right skewed nature of the dependent variable and possible heteroskedasticity. Should administrative censoring (due to patients differential follow-up) be present, the analysis will use inverse probability weighting (IPW) methods alongside our cost regression models.[38]

All regression models will explore the impact of the patient's baseline characteristics included in Table 1, and use these results to explore the role of patients' heterogeneity onto the cost-effectiveness results (more in section 3.2.3).

3.1.3 Sensitivity analysis

For the purposes of the CCA, we will carry out a per protocol analysis and compare the results from the ITT. We will calculate the minimum vs actual number of group sessions needed to provide the I-WOTCH intervention to all participants in the intervention group. We will also consider a minimum and maximum number of participants per course observed in the I-WOTCH trial. We will use a one-way or threshold analyses on the assumptions made on estimating the costs.

3.2 Long-term model-based cost-effectiveness analysis

3.2.1 Populating model parameters

Table 2 provides a list of key model parameters and the source for initial values, i.e. mean, SD or confidence interval. To estimate all model initial values we will use IPD from the I-WOTCH

For peer review only - http://bmjopen.bmj.com/site/about/guidelines.xhtml

trial and GP records as well as any relevant publicly available evidence. Transitions from all model states to death will be extracted from age and sex adjusted all-cause mortality statistics from the ONS.[39] Transition probabilities between all the other model states will be derived using risk equations from regression models (e.g. time-to-event and/or logit regression models depending on the nature of the transition). Costs and utilities parameters for each of the model states and events will be estimated using the regression models described in section 3.1.2. Variance-covariance matrices for each of the regressions will be extracted to inform the parameter uncertainty estimates in the model and used in the probabilistic sensitivity analysis (PSA) as explained in section 3.2.4 below.

Table 2. Model parameters to inform long-term decision analytic model

Transition probability	***Sources***	***Specific details of the fields in source***
Remaining in IT	NTP<br>NCRF	Time of withdrawal from IT<br>Time of withdrawal from IT
LTOT to IT	The trial	Proportion of people engaged in IT
IT to LTOT	NTP<br>NCRF	Time of withdrawal from IT<br>Time of withdrawal from IT
IT to SOLMP	NTP<br>NCRF	Time of completely stopping the use of opioids<br>Time of completely stopping the use of opioids
IT to dead state	PL	All-cause mortality data from ONS
Remaining in LTOT	The trial	Number of people who do not engage in IT and remain in LTOT
SOLMP to LTOT	PtQ<br>GPRs	Medication data<br>Prescription data
LTOT to PT	PtQ<br>GPRs	Medication data<br>Prescription data
LTOT to dead state	PL	All-cause mortality data from ONS
Remaining in PT	The trial<br>PtQ<br>GPRs	Number of people who remain in PT over time<br>Medication data<br>Prescription data
PT to LTOT	PtQ<br>GPRs	Medication data<br>Prescription data
PT to SOLMP	PtQ<br>GPRs	Medication data<br>Prescription data
PT to dead state	PL	All-cause mortality data from ONS
Remaining in SOLMP	PtQ<br>GPRs	Medication data<br>Prescription data
SOMP to dead state		All-cause mortality rates from ONS
Utility scores	***Source***	***Specific details of the source***
LTOT	PL<br>PtQ	Utility of opioid therapy [40]<br>Regression analysis of patient self-reported EQ-5D
PT	PtQ	Regression analysis of patient self-reported EQ-5D

For peer review only - http://bmjopen.bmj.com/site/about/guidelines.xhtml

IT	PtQ	Regression analysis of patient self-reported EQ-5D
SOLMP	PtQ	Regression analysis of patient self-reported EQ-5D
Costs	***Source***	***Specific details of the source***
LTOT	PL<br>PtQ<br>GPRs	Cost of opioid therapy per cycle [41]<br>Costs associated with self-reported resource use<br>Costs associated with resource use collected
PT	The trial<br>PtQ<br>GPRs	Cost associated with usual care<br>Costs associated with self-reported resource use<br>Costs associated with resource use collected
IT	The trial	Cost associated with intervention
SOLMP	PL	Assumption - 1x contact with GP [28]
Key for Table 2: GPRs - GP records, IT - I-WOTCH tapering, LTOT - Long-term opioid therapy, NCRF -nurse clinical record form, NTP - Nurse tapering plan, PL - Published literature, PT - Patient driven tapering, PtD - Patient diaries, PtQ - Patient questionnaire, SOLMP - Successful opioid-less management of pain, The trial - I-WOTCH trial		

3.2.2 Incremental analysis

We will estimate the differential costs and QALYs predicted by the model and - where relevant - calculate the incremental cost-effectiveness ratio (ICER), defined as the ratio between the mean difference in costs and the mean difference in QALYs. As decision makers need to assess whether an intervention is 'value for money' we will compare the ICER against two 'thresholds': one ranging between £20,000 and £30,000 per QALY gained to mimic the criterion used by NICE for its policy decisions, and another recently proposed by Lomas *et al.* who estimated this threshold to be between £5,000 and £15,000.[42]

3.2.3 Subgroup analysis

In order to reflect variation between individuals in terms of their health benefit, resource use, and costs, we will run the Markov state transition model for a series of different patient profiles. This will enable us to reflect the impact that patient characteristics may have on the value for money assessment of I-WOTCH. The rationale for this approach stems from the recognition that the baseline risk (and possibly the treatment effect) may vary between individuals based on their opioid use, pain severity, and pain conditions. The trial's pre-defined subgroups based on baseline variables are given below:

i) pain severity score (5 to 8, 9 to 11, and 12 to 15);
ii) opioid usage in MED (0 -20 mg, 30-59 mg, 60 -89 mg, 90-119 mg, 120 -149 mg, and >= 150 mg); and
iii) pain conditions (fibromyalgia, musculo-skeletal, arthritis, back pain, neurological, cancer, and other), and
iv) duration of opioid intake (Less than 1 year, 1-5 years, and more than 5 years).

For peer review only - http://bmjopen.bmj.com/site/about/guidelines.xhtml

3.2.4 Probabilistic sensitivity analyses

Sampling uncertainty in the model will be captured by characterising each model parameter with an appropriate probability distribution. We will derive the parameters to inform these distributions from the variance-covariance matrix obtained from each regression model. Cholesky decomposition of each variance-covariance matrix will be used to make the simulation more efficient. Sampling uncertainty will be propagated through the model using Monte-Carlo simulation in the form of PSA to understand the effect on the predicted mean QALYs and mean costs associated with each treatment arm. These parameters will then be combined and compared against a range of feasible cost-effectiveness thresholds to obtain an estimate of the probability that I-WOTCH is cost-effective. This information will be represented graphically in the form of a cost-effectiveness acceptability curve (CEAC).[43] Also, we will perform scenario analyses for the various distributional assumptions made, e.g. on the parametric survival distribution and compare the results for different assumptions.

4. Discussion

The scale of the opioid crisis in the UK is not as severe as in the United States of America. However, recently, it has been reported that opiates are a frequent cause of death due to drug poisoning in the UK.[3,44] To begin reviewing the benefits and risks of opioid medicine, and make recommendations for regulatory action, an expert working group has been formed by the Medicines and Healthcare Products Regulatory Agency (MHRA).[45] This protocol describes the first economic evaluation of an adjunct intervention (i.e. I-WOTCH) to support opioid tapering in patients with chronic non-malignant pain. Its findings will provide timely and significant results to inform policy recommendations on how best to tackle the opioids epidemic in the UK and manage the complex landscape of opioids related health (and financial) risks.

A study that tested the effectiveness of an earlier version of I-WOTCH (i.e. the COPERS trial COping with persistent Pain, Effectiveness Research into Self-management) showed sustained benefits on depression and anxiety in people with musculoskeletal chronic pain.[46] The COPERS intervention was designed based on cognitive behavioural therapy principles. While in the COPERS trial more than 20% of participants used strong opioids this was not aimed at opioid dose reduction. In contrast, the I-WOTCH intervention was designed to support opioid reduction and improve activities of daily living.

4.1 Strengths

This paper describes the first economic evaluation analysis of a complex intervention to support opioid tapering, which uses data collected alongside the first UK based RCT. A within-trial CCA will allow estimating relevant model parameters. The long-term impact on costs and health benefits from implementing the I-WOTCH programme in England will be assessed in a lifetime decision analytic model populated using trial and previously published data. This analysis is based on a robust conceptual model that reflects clinical practice and long-term adverse events associated with both opioid use and opioid withdrawal. This model will enable estimation of I-WOTCH's cost-effectiveness over a lifetime horizon and real-life subgroup analyses.

For peer review only - http://bmjopen.bmj.com/site/about/guidelines.xhtml

4.2 Limitations

Our decision problem and associated model structure are complex. I-WOTCH's effectiveness in the short and long term is associated to the occurrence/absence of several interlinked events that determine individual's transition from one health state to the other. I-WOTCH's 12-month trial follow-up period is unlikely to allow to capture all events of interest in the long-term. This may limit our ability to estimate all relevant transition probabilities on trial based IPD. A number of model parameters may need to be estimated from published sources and experts' opinion. Data availability may limit the successful evaluation of the CEA model.

Ethics and dissemination

The study was approved by the Yorkshire & The Humber – South Yorkshire Research Ethics Committee (16/YH/0325). Appropriate local approvals were sought for each area in which recruitment was undertaken. The current protocol version is 1.7 date 31 July 2019. The trial is registered with an International Standard Randomised Controlled Trial Number (ISRCTN) Register. ISRTC number 49470934 (6 Feb 2017). To inform all health technology assessment stakeholders our results will be published in peer reviewed journals and presented at scientific conferences. Similarly, I-WOTCH's newsletter and lay summaries of our results on the study's website will be our main vehicles to disseminate our findings to study participants and facilitators.

Patient and public involvement

The Involvement of patient and public in the intervention design, development, and delivery are outlined in the I-WOTCH clinical protocol paper.[19] In brief, two lay advisers with chronic pain withdrawal of opioids and experience of clinical trial research have been recruited to the study. Additionally, prior to receiving funding for the study, meetings were held with volunteers with chronic malignant pain to receive input to the design of the intervention structure, duration of intervention and content to be covered. These meeting contributed also to the design of the study including randomisation, best usual care intervention, recruitment processes as well as outcome measures.

Acknowledgements

The authors would like to thank members of the I-WOTCH trial team for their comments on previous versions of I-WOTCH economic analysis plan and acknowledge the inputs of the investigators contributing to the conduct of the trial.

I-WOTCH team

(Chief investigator) - Harbinder Kaur Sandhu, Warwick Clinical Trials Unit, University of Warwick

(Co- Chief investigator) - Sam Eldabe, The James Cook University Hospital, Cheriton House, Marton Road Middlesbrough

For peer review only - http://bmjopen.bmj.com/site/about/guidelines.xhtml

For peer review only

Charles Abraham, School of Psychological Sciences, Faculty of Medicine, Dentistry and Health Sciences, University of Melbourne, Victoria 3010, Australia

Sharisse Alleyne, Warwick Clinical Trials Unit, University of Warwick

Shyam Balasubramanian, Department of Anaesthesia & Pain Medicine University Hospitals Coventry and Warwickshire NHS Trust

Lauren Betteley, Warwick Clinical Trials Unit, Warwick Medical School

Katie Booth, Warwick Clinical Trials Unit, University of Warwick

Dawn Carnes, Barts & The London Queen Mary's School of Medicine & Dentistry

Andrea Dompieri Furlan, Toronto Rehabilitation Institute, University Health Network, Canada.

Kirstie Haywood, Division of Health Sciences, University of Warwick.

Maddy Hill, Warwick Clinical Trials Unit, University of Warwick

Ranjit Lall, Warwick Clinical Trials Unit University of Warwick.

Andrea Manca, Centre for Health Economics, University of York

Dipesh Mistry, Warwick Clinical Trials Unit, University of Warwick.

Vivien P Nichols, Warwick Clinical Trials Unit, Warwick Medical School, University of Warwick,

Jennifer Noyes, The James Cook University Hospital, Cheriton House, Marton Road Middlesbrough

Anisur Rahman, Centre for Rheumatology Research, University College London, London

Kate Seers, Warwick Research in Nursing, Warwick Medical School, University of Warwick,

Jane Shaw, The James Cook University Hospital, Cheriton House, Marton Road Middlesbrough

Nicole Kit Yee Tang, Department of Psychology University of Warwick.

Stephanie Taylor, Barts & The London Queen Mary's School of Medicine & Dentistry.

Colin Tysall, University/User Teaching and Research Action Partnership, University of Warwick

Martin Underwood Warwick Clinical Trials Unit, University of Warwick, Coventry

Emma Withers, Warwick Clinical Trials Unit, University of Warwick.

Cynthia Paola Iglesias Urrutia, Centre for Health Economics, University of York

Authors' contributions

SMK drafted the first version of this manuscript, CPIU is responsible for overseeing the design and implementation of the I-WOTCH economic analysis and for drafting subsequent versions of the manuscript, VSG contributed to the review of the literature of economic evaluation studies, AM provided expert advice and contributed to the draft of the manuscript. HS and SE are Co-Chief Investigators and oversee the running of the trial. MU has provided input into all aspects of the trial study design and support in running of the study. SMK, VSG, CPIU, HS, MU, SE, and AM reviewed and approved the final version of the paper.

For peer review only - http://bmjopen.bmj.com/site/about/guidelines.xhtml

Funding statement

This project is funded by the National Institute of Health Research (NIHR), Health Technology Assessment (HTA) (project number 14/224/04). The views and opinions expressed therein are those of authors and do not necessarily reflect those of HTA, NIHR, or NHS.

Competing interests

MU was the Chair of the NICE accreditation advisory committee until March 2017 for which he received a fee. He is the chief investigator or co-investigator on multiple previous and current research grants from the UK National Institute for Health Research, Arthritis Research UK, and is a co-investigator on grants funded by the Australian NHMRC. MU is an NIHR Senior Investigator and has received travel expenses for speaking at conferences from the professional organisations hosting the conferences. He is a director and shareholder of Clinvivo Ltd. that provides electronic data collection for health services research. MU is part of an academic partnership with Serco Ltd. related to return to work initiatives. He is a co-investigator on a study receiving support in kind from Stryker Ltd. MU has accepted honoraria for teaching/lecturing from the consortium for advanced research training in Africa. He is an editor of the NIHR journal series, and a member of the NIHR Journal Editors Group, for which he receives a fee. SE is an investigator on number of NIHR and industry-sponsored studies. SE received travel expenses for speaking at conferences from the professional organisations organising these conferences. SE attended advisory boards and provided consultancy services for Medtronic, Abbott, Boston Scientific, and Mainstay Medical, none in relation to opioids. SE's department has received research funding from Medtronic. HS is the director of Health Psychology Services Ltd, providing psychological services for a range of health-related conditions. AM has received consultancy fees for participating in Pharmaceutical and Medical Device Industry Advisory Boards in the area of musculoskeletal pain. AM is a member of the NICE Technology Appraisal Committee. CPIU is a member of the NICE Medical Technologies Advisory Committee.

For peer review only

References

1. Fayaz A, Croft P, Langford RM, et al. Prevalence of chronic pain in the UK: a systematic review and meta-analysis of population studies. *BMJ Open* 2016;6(6):e010364.
2. Curtis HJ, Croker R, Walker AJ, et al. Opioid prescribing trends and geographical variation in England, 1998-2018: a retrospective database study. *Lancet Psychiatry* 2019;6(2):140-50.
3. Taylor S, Annand F, Burkinshaw P, et al. Dependence and withdrawal associated with some prescribed medicines: an evidence review. *London: Public Health England* 2019. https://assets.publishing.service.gov.uk/government/uploads/system/uploads/attachment_data/file/829777/PHE_PMR_report.pdf (accessed on December 16, 2019).
4. Manchikanti L, Ailinani H, Koyyalagunta D, et al. A systematic review of randomized trials of long-term opioid management for chronic non-cancer pain. *Pain Physician* 2011;14(2):91-121.
5. Noble M, Treadwell JR, Tregear SJ, et al. Long-term opioid management for chronic noncancer pain. *Cochrane Database Syst Rev* 2010(1):CD006605.
6. Els C, Jackson TD, Kunyk D, et al. Adverse events associated with medium- and long-term use of opioids for chronic non-cancer pain: an overview of Cochrane Reviews. *Cochrane Database of Systematic Reviews* 2017(10)
7. Warner M, Trinidad JP, Bastian BA, et al. Drugs most frequently involved in drug overdose deaths: United States, 2010–2014. *Natl Vital Stat Rep* 2016
8. Moore RA, McQuay HJ. Prevalence of opioid adverse events in chronic non-malignant pain: systematic review of randomised trials of oral opioids. *Arthritis Res Ther* 2005;7(5):R1046-51.
9. Sullivan MD, Edlund MJ, Zhang L, et al. Association between mental health disorders, problem drug use, and regular prescription opioid use. *Archives of Internal Medicine* 2006;166(19):2087-93.
10. Darcq E, Kieffer BL. Opioid receptors: drivers to addiction? *Nature Reviews Neuroscience* 2018;19(8):499-514.
11. Kim JY, Kim JH, Yee J, et al. Risk factors of opioid-induced adverse reactions in elderly male outpatients of Korea Veterans Hospital. *Bmc Geriatrics* 2018;18
12. Bedson J, Chen Y, Ashworth J, et al. Risk of adverse events in patients prescribed long-term opioids: A cohort study in the UK Clinical Practice Research Datalink. *Eur J Pain* 2019;23(5):908-22.
13. Dowell D, Haegerich TM, Chou R. CDC Guideline for Prescribing Opioids for Chronic Pain--United States, 2016. *JAMA* 2016;315(15):1624-45.
14. Sandhu H, Underwood M, Furlan AD, et al. What interventions are effective to taper opioids in patients with chronic pain? *Bmj-British Medical Journal* 2018;362
15. Gowing L, Ali R, White JM. Buprenorphine for the management of opioid withdrawal. *Cochrane Database of Systematic Reviews* 2009(3)
16. Frank JW, Lovejoy TI, Becker WC, et al. Patient Outcomes in Dose Reduction or Discontinuation of Long-Term Opioid Therapy A Systematic Review. *Annals of Internal Medicine* 2017;167(3):181-+.
17. Gowing L, Ali R, White JM. Opioid antagonists with minimal sedation for opioid withdrawal. *Cochrane Database of Systematic Reviews* 2017(5)

For peer review only - http://bmjopen.bmj.com/site/about/guidelines.xhtml

18. Sullivan MD, Turner JA, DiLodovico C, et al. Prescription Opioid Taper Support for Outpatients With Chronic Pain: A Randomized Controlled Trial. *Journal of Pain* 2017;18(3):308-18.
19. Sandhu HK, Abraham C, Alleyne S, et al. Testing a support programme for opioid reduction for people with chronic non-malignant pain: the I-WOTCH randomised controlled trial protocol. *BMJ Open* 2019;9(8):e028937.
20. National Institute for Health and Clinical Excellence. Guide to the methods of technology appraisal 2013 [Available from: https://www.nice.org.uk/process/pmg9/resources/guide-to-the-methods-of-technology-appraisal-2013-pdf-2007975843781 (accessed on December 16, 2019)].
21. A brief guide to the PROMIS© Pain Interference instruments [Available from: http://www.healthmeasures.net/images/PROMIS/manuals/PROMIS_Pain_Interference_Scoring_Manual.pdf(date (accessed on December 16, 2019)].
22. Vernon MK, Reinders S, Mannix S, et al. Psychometric evaluation of the 10-item Short Opiate Withdrawal Scale-Gossop (SOWS-Gossop) in patients undergoing opioid detoxification. *Addictive Behaviors* 2016;60:109-16.
23. Herdman M, Gudex C, Lloyd A, et al. Development and preliminary testing of the new five-level version of EQ-5D (EQ-5D-5L). *Quality of Life Research* 2011;20(10):1727-36.
24. Position statement on use of the EQ-5D-5L value set for England [Available from: https://www.nice.org.uk/about/what-we-do/our-programmes/nice-guidance/technology-appraisal-guidance/eq-5d-5l (accessed on December 16, 2019)].
25. Devlin NJ, Shah KK, Feng Y, et al. Valuing health-related quality of life: An EQ-5D-5L value set for England. *Health Econ* 2018;27(1):7-22.
26. van Hout B, Janssen MF, Feng YS, et al. Interim scoring for the EQ-5D-5L: mapping the EQ-5D-5L to EQ-5D-3L value sets. *Value Health* 2012;15(5):708-15.
27. Improvement NHS. Reference costs 2018/19: highlights, analysis and introduction to the data 2019 [Available from: https://improvement.nhs.uk/resources/national-cost-collection/#ncc1819 (accessed on May 27, 2020)].
28. Curtis L, Burns A. Unit Costs of Health and Social Care 2019 [Available from: https://www.pssru.ac.uk/project-pages/unit-costs/unit-costs-2019/ (accessed on May 27, 2020)].
29. Curtis L. Unit costs of health and social care 2013 [Available from: http://www.pssru.ac.uk/project-pages/unit-costs/2013/ (accessed on December 16, 2019)].
30. Curtis L, Burns A. Unit Costs of Health and Social Care 2018 [Available from: https://www.pssru.ac.uk/project-pages/unit-costs/unit-costs-2018/ (accessed on December 16, 2019)].
31. Inflation and price indices 2019 [Available from: https://www.ons.gov.uk/economy/inflationandpriceindices (accessed on December 16, 2019)].
32. Joint Formulary Committee. British national formulary, 77th Edition 2019.
33. R : A Language and Environment for Statistical Computing [program], 2014.
34. Faria R, Gomes M, Epstein D, et al. A guide to handling missing data in cost-effectiveness analysis conducted within randomised controlled trials. *Pharmacoeconomics* 2014;32(12):1157-70.

For peer review only - http://bmjopen.bmj.com/site/about/guidelines.xhtml

35. Wailoo AJ, Hernandez-Alava M, Manca A, et al. Mapping to Estimate Health-State Utility from Non-Preference-Based Outcome Measures: An ISPOR Good Practices for Outcomes Research Task Force Report. *Value Health* 2017;20(1):18-27.
36. Hernandez M, Wailoo A, Pudney S, et al. Modelling generic preference based outcome measures - development and comparison of methods. *Health Technology Assessment* 2019
37. Mihaylova B, Briggs A, O'Hagan A, et al. Review of Statistical Methods for Analysing Healthcare Resources and Costs. *Health Economics* 2011;20(8):897-916.
38. Lin DY. Linear regression analysis of censored medical costs. *Biostatistics* 2000;1(1):35-47.
39. Office for National Statistics. Deaths registered in England and Wales [Available from: https://www.ons.gov.uk/peoplepopulationandcommunity/birthsdeathsandmarriages/deaths (accessed on December 17, 2019)].
40. Ikenberg R, Hertel N, Moore RA, et al. Cost-effectiveness of tapentadol prolonged release compared with oxycodone controlled release in the UK in patients with severe non-malignant chronic pain who failed 1st line treatment with morphine. *J Med Econ* 2012;15(4):724-36.
41. Sullivan W, Hirst M, Beard S, et al. Economic evaluation in chronic pain: a systematic review and de novo flexible economic model. *Eur J Health Econ* 2016;17(6):755-70.
42. Lomas J, Martin S, Claxton K. Estimating the Marginal Productivity of the English National Health Service From 2003 to 2012. *Value Health* 2019;22(9):995-1002.
43. Fenwick E, O'Brien BJ, Briggs A. Cost-effectiveness acceptability curves--facts, fallacies and frequently asked questions. *Health Econ* 2004;13(5):405-15.
44. John E, Butt A, McQuade G. Deaths related to drug poisoning in England and Wales: 2018 registrations. https://www.ons.gov.uk/peoplepopulationandcommunity/birthsdeathsandmarriages/deaths/bulletins/deathsrelatedtodrugpoisoninginenglandandwales/2018registrations#links-to-related-ons-information (accessed on January 21, 2019).
45. Opioid Expert Working Group meets at MHRA [Available from: https://www.gov.uk/government/news/opioid-expert-working-group-meets-at-mhra (accessed on December 12, 2019)].
46. Taylor SJ, Carnes D, Homer K, et al. Novel Three-Day, Community-Based, Nonpharmacological Group Intervention for Chronic Musculoskeletal Pain (COPERS): A Randomised Clinical Trial. *PLoS Med* 2016;13(6):e1002040.

For peer review only

List of abbreviations

ADL - Activities of Daily Living

BNF - British National Formulary

CCA - Cost-Consequences Analysis

CEA - Cost-Effectiveness Analysis

CEAC - Cost-Effectiveness Acceptability Curve

COPERS - COping with persistent Pain, Effectiveness Research into Self-management

D - Death

GLM - Generlised Linear Models

GP - General Practitioner

HRG - Healthcare Resource Group

HTA - Health Technology Assessment

ICER - Incremental Cost-Effectiveness Ratio

IPD – Individual Patient Data

IPW - Inverse Probability Weighting

ISRCTN - International Standard Randomised Controlled Trial Number

ITT - Intention To Treat

IT - I-WOTCH Tapering

I-WOTCH - Improving the Wellbeing of people with Opioid Treated CHronic Pain

LTOT - Long-Term Opioid Therapy

MHRA - Medicines and Healthcare products Regulatory Agency

NHS - National Health Service

NICE - National Institute for Health and Care Excellence

NIHR - National Institute of Health Research

For peer review only - http://bmjopen.bmj.com/site/about/guidelines.xhtml

MED - Morphine Equivalent Dose

ONS - Office of National Statistics

PROMIS-PI-SF-8A - Patient Reported Outcomes Measurement Information System Pain Intensity Short Form-8A

PSA - Probabilistic Sensitivity Analysis

PSAE - Persistent/Serious Adverse Events

PSSRU - Personal and Social Services Research Unit

PT - Patient-driven Tapering

QALY - Quality Adjusted Life Year

RCT - Randomised Controlled Trial

SD - Standard Deviation

ShOWS - Short Opiate Withdrawal Scale

SOLMP - Successful Opioid-Less Management of Pain

TEAE - Transient/Emergent Adverse Events

For peer review only

For peer review only - http://bmjopen.bmj.com/site/about/guidelines.xhtml

Figure captions

Figure 1: Simplified model structure showing patient flow during the I-WOTCH trial. At any time, individuals will be allocated to any five health states (shown as ovals). Individuals start in the long-term opioid therapy (LTOT) health state. Depending on the trial arm, they will move to either I-WOTCH tapering (IT) or patient-driven tapering (PT). The transition from LTOT to IT occurs only at the start of the trial and patients cannot go back to IT once they withdraw from it. Patients who have tapered their opioid doses completely will be in the successful opioid less management of pain (SOLMP) health state. Patients may withdraw from IT or PT to move to LTOT health state. Similarly, patients in SOLMP may move to LTOT if they restart their opioid doses. The arrows' spearheads indicate the direction of allowed transition from one state to the other. Dead (D) state is an absorbing state where no transitions from the dead state are allowed.

For peer review only

For peer review only - http://bmjopen.bmj.com/site/about/guidelines.xhtml

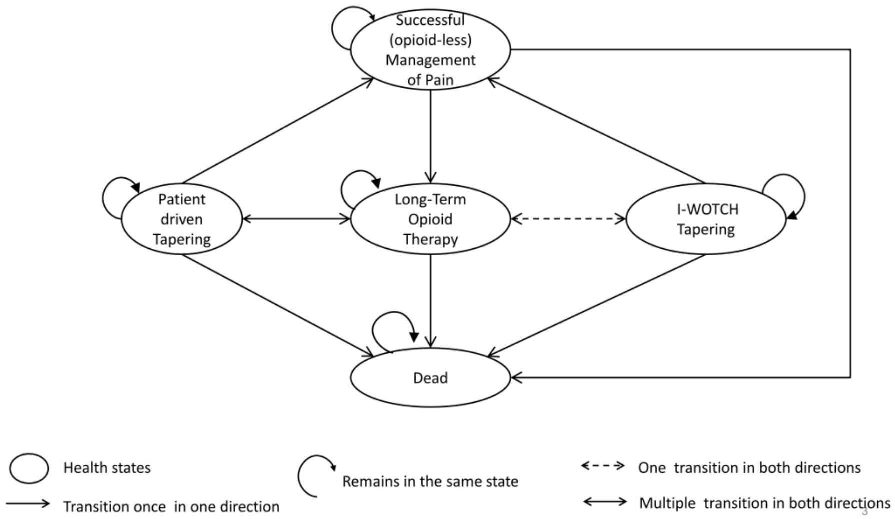

Figure 1: Simplified model structure showing patient flow during the I-WOTCH trial. At any time, individuals will be allocated to any five health states (shown as ovals). Individuals start in the long-term opioid therapy (LTOT) health state. Depending on the trial arm, they will move to either I-WOTCH tapering (IT) or patient-driven tapering (PT). The transition from LTOT to IT occurs only at the start of the trial and patients cannot go back to IT once they withdraw from it. Patients who have tapered their opioid doses completely will be in the successful opioid less management of pain (SOLMP) health state. Patients may withdraw from IT or PT to move to LTOT health state. Similarly, patients in SOLMP may move to LTOT if they restart their opioid doses. The arrows' spearheads indicate the direction of allowed transition from one state to the other. Dead (D) state is an absorbing state where no transitions from the dead state are allowed.

338x190mm (300 x 300 DPI)

Supplementary Material

Resource use and costing for the I-WOTCH Intervention

**Table A1. Resource use and costs related to running I-WOTCH intervention**		
**I-WOTCH intervention running costs**		
Category	***Resource use***	***Unit cost***
Number of participants	Number of participants enrolled in the course	
Facilitator costs	Total number and duration of sessions per facilitator	Fixed fee per session (pension and national Insurance contributions included). Alternatively, grade (if NHS) or salary
Facilitator travel costs	Total number and type (i.e. car, train, etc.) of journeys	Train or bus fare and fixed fee per mile.
Administrator costs	Number of days / months dedicated to coordinate implementation of I-WOTCH intervention	Salary used to estimate fixed daily rate (pension and national insurance contributions included)
Facility costs	Number of days facility has been hired per course delivered	Daily rate for venue
Hospitality costs	Food and drink	
Consumables	Stationary (e.g. letters, stamps, notebooks, pens, pencils, etc.)	
Course materials costs		
My Opioid Manager Manual	Total number of manuals produced and delivered	Total printing costs and copy right fee
Relaxation Package	Total number of CDs produced and delivered	Total cost of CDs

For peer review only

Table A2. Resource use and costs related to training related to I-WOTCH intervention		
**Training costs**		
Category	***Resource use***	***Unit cost***
Trainer costs	Number of days / months - per trainer - dedicated to train facilitators to deliver I-WOTCH intervention	Fixed daily rate
Trainer travel cost	Total number and type (i.e. car, train, etc.) of journeys	Train or bus fare and fixed fee per mile
Facilitator costs	Total number and duration of sessions per facilitator	Fixed fee per session (pension and national insurance contributions included) or salary
Facilitator travel costs	Total number and type (i.e. car, train, etc.) of journeys	Train or bus fee and fixed fee per mile
Administrator costs	Number of days / months dedicated to coordinate implementation of I-WOTCH intervention	Salary used to estimate fixed daily rate (pension and national insurance contributions included)
Facility costs	Number of days facility has been hired per course delivered	Daily rate for venue
Hospitality costs		
Consumables		
Course materials costs		
My Opioid Manager Manual	Total number of manuals produced and delivered	Total printing costs
Relaxation Package	Total number of CDs produced and delivered	Total cost of CDs

For peer review only

**Table A3. Data collection form used to characterise the I-WOTCH intervention facilitators**	
**Characteristics of course facilitators**	
Variable	***Description***
Facilitator ID	Define unique key
Site	
Age	In years
Gender	
Ethnicity	Same categories used for participants
Type	Healthcare provider<br>Lay person
Profession / Experience	
Years of professional experience (for health care professional (HCP)) or facilitation (lay person)	
Number of curses facilitated	

**Table A4. Data collection form used to characterise the course in I-WOTCH intervention**	
**Course venues, facilitators, and participants**	
Variable	***Description***
Course ID	
Venue	
Facilitator	Facilitator profession/experience
Number enrolled	
Attendance	Attendance per patient per session
